# CENPT prevents renal cell carcinoma against ferroptosis by enhancing the synthesis of glutathione

**DOI:** 10.1038/s41419-025-07848-x

**Published:** 2025-07-12

**Authors:** Han Yang, Zongliang Zhang, Ninghan Feng, Kai Zhao, Yulian Zhang, Xinbao Yin, Guanqun Zhu, Zhenlin Wang, Xuechuan Yan, Xueyu Li, Zhaofeng Li, Qinglei Wang, Yixin Qi, Peng Zhao, Tianzhen He, Ke Wang

**Affiliations:** 1 Department of Urology, The Affiliated Hospital of Qingdao University, Qingdao, China; 2 Department of Urology, Jiangnan University Medical Center, Wuxi, China; 3 Department of Urology, Wuxi No.2 Hospital, Nanjing Medical University, Wuxi, China; 4 Department of Gynecology, The Affiliated Hospital of Qingdao University, Qingdao, China; 5 Faculty of Sport Science and Coaching, Universiti Pendidikan Sultan Idris, Tanjong Malim, Perak Darul Ridzuan Malaysia; 6 Nantong University, Institute of special environmental medicine, Nantong, China

**Keywords:** Tumour biomarkers, Prognostic markers

## Abstract

Cancer is characterized by chromosomal instability (CIN), which leads to tumor heterogeneity and other malignant features. CIN is caused by abnormal centromere and kinetochore function, which results in aneuploidy, rearrangements, and micronucleus production. Centromere and kinetochore gene misexpression plays a vital role in tumor progression. Here we show that Centromere Protein T (CENPT) is highly expressed in renal carcinoma (RCC) and promotes the tumor proliferation and metastasis of RCC. CENPT is found to be critical for regulating the glutathione (GSH) metabolism pathway because it interacts with γ-glutamyl-cysteine ligase catalytic subunit (GCLC), consequently reducing reactive oxygen species levels and inhibiting ferroptosis. Mechanistically, CENPT increases the catalytic activity of GCLC by directly binding to GCLC ∆213-424aa competitively with glutamate-cysteine ligase modifier subunit (GCLM), consequently induces the GSH synthesis. In turn, GSH increases CENPT expression via transcriptional regulation mediated by the transcription factor ATF2, forming a CENPT-GCLC-GSH feedback loop that enhances the pro-carcinogenic effect of this axis in RCC. Our study identifies CENPT a potential target for RCC via forming a CENPT-GCLC-GSH feedback loop to inhibit ferroptosis. This may support a promising treatment strategy for RCC.

## Introduction

Renal cell carcinoma (RCC) is one of the top ten most common malignancies worldwide and the second leading cause of death in patients with urologic tumors [1]. Moreover, the incidence and mortality rates of RCC have been increasing year by year in the last two decades of statistics worldwide [2]. According to different histological types, RCC is largely classified into three subtypes, including papillary RCC (pRCC), clear cell RCC (ccRCC) and chromophobe RCC (chRCC). Malignancies of these three subtypes exhibit their different specific cytogenetic characteristics, different prognosis and treatment response, among which ccRCC is the most common, accounting for about 75% [3]. The insidious nature of its development allows more than one-third of RCC patients to have distant metastases at the time of initial presentation. In recent years, targeted therapies and immunotherapy for kidney cancer have improved overall survival, but most patients eventually develop resistance to radiotherapy, chemotherapy and targeted agents, and 5-year survival rates for patients with advanced RCC are extremely low [4]. Therefore, it is imperative to explore reliable and effective biomarkers, as well as potential therapeutic targets for RCC.

Chromosomal instability (CIN), a hallmark of cancer, leads to tumor heterogeneity and other malignant features [5, 6]. CIN is caused by abnormal centromere and kinetochore function, which results in aneuploidy, rearrangements, and micronucleus production [7]. Centromere and kinetochore gene misexpression plays a vital role in tumor progression [8]. In addition, CIN induction leads to sensitivity to metabolic stress [9]. Because CIN cells are already under stress, a small metabolic disturbance that does not impact normal cells might result in significant levels of oxidative stress and consequent cell death [9]. Changed metabolism is a distinguishing feature of cancer cells [9]. The involvement of centromere and kinetochore genes, which produced CIN in cancer cells, in modifying cancer cell metabolism must therefore be further investigated.

Glutathione (GSH) is the most prevalent antioxidant present in living creatures and has a variety of activities, the majority of which are related to cellular redox equilibrium [10]. GSH is a tripeptide composed of glutamic acid, cysteine and glycine [11]. It is widely expressed in various types of cells and plays an important antioxidant and detoxification function. In recent years, studies have revealed an increasing interest in the role of GSH in tumors, especially in the mechanisms of regulation and effects in a variety of tumor types [12]. The role of GSH in tumors is mainly achieved through its antioxidant and detoxification capacity. Tumor cells are usually in a state of high oxidative stress, generating large amounts of free radicals and oxidants, leading to intracellular oxidative damage [13]. GSH, as an important antioxidant, can neutralize free radicals and oxidants and maintain intracellular redox balance, thus protecting tumor cells from oxidative damage [14]. In addition to its antioxidant effect, GSH is also involved in regulating cell proliferation, apoptosis and modulating the tumor microenvironment, among other processes [15].

The γ-glutamyl cycle, first proposed in 1970, contains enzymes that depend on both γ-glutamylcysteine synthetase (GCL) and glutathione synthetase (GSS), as well as γ-glutamyl transferases (GGTs) for the entire process of GSH biosynthesis and degradation, respectively [16]. GCL, consists of two distinct subunits: the GCLC catalytic subunit, which contains the active site of the catalytic reaction, and the regulatory subunit GCLM, which interacts with GCLC to improve the catalytic efficiency of the regulatory subunit GCLM. It is involved in the first step of glutathione biosynthesis and is the rate-limiting enzyme, which catalyzes the γ-glutamyl cysteine production reaction [17]. Increased protein binding of GCLC and GCLM has been reported to cause a significant increase in enzyme activity in ccRCC tissues [18–21]. Moreover, it was reported that GCLC silencing mediated by shRNA resulted in a significant decrease in the number of cells in RCC cell lines, along with a significant decrease in the level of GSH expression [14, 19]. These data support the important role of GSH metabolism in RCC development and progression.

The primary mechanism underlying ferroptosis involves the induction of intracellular ROS production and disruption of the balance in degradation. Ferroptosis induction is predominantly achieved through direct or indirect modulation of glutathione peroxidase, resulting in diminished cellular antioxidant capacity, accumulation of ROS, and eventual initiation of oxidative cell death [22]. Increased synthesis of GSH has been shown to block ROS-induced stress signal transduction, thereby inhibiting ferroptosis in tumor cells [23, 24].

In the present study, we found that centromere and kinetochore gene, CENPT, functions as a regulator of GSH synthesis and then inhibits ferroptosis of RCC cells in vitro and in vivo. Through CO-IP assays, we also observed that intracellular CENPT directly competitively binds with GCLC to induce the synthesis of GSH, thereby inhibiting ferroptosis in RCC. In turn, GSH increases the expression of CENPT, forming a CENPT-GCLC-GSH feedback loop to enhance the pro-carcinogenic effect of CENPT-GCLC-GSH axis on RCC. Taken together, CENPT may play a tumorigenic role in the progression of RCC and may be a new potential prognostic biomarker and therapeutic target.

## Methods

### Patient tissue specimens

Tissue samples were collected from RCC patients diagnosed at the Department of Urology, Affiliated Hospital of Qingdao University, China. This study followed the guiding principles of the Declaration of Helsinki and was approved by the Ethics Committee of the Affiliated Hospital of Qingdao University (Qingdao, China, QYFYWZLL 26556). All patients were aware of their specimen content, potential risks and the purpose of the study, and had signed a written informed consent. RNA was extracted from 10 pairs of frozen RCC tumor tissues and adjacent non-tumor tissues for quantitative real-time fluorescence quantitative PCR (RT-qPCR) assay and quantification of total RNA CENPT levels.

### RCC cell culture

Human RCC lines (ACHN, OSRC-2, A498, ORSC-2) and human renal epithelial cell line HK2 were obtained from Gong Kan lab, Institute of Urology, Peking University, while RCC lines (786-O and caki-1) were purchased from Wuhan Pricella Biotechnology Co., Ltd, China. All cell lines were recently authenticated by STR profiling and tested negative for mycoplasma contamination. Human RCC cell lines were cultured in DMEM medium (PM150210, Pricella, China) containing 10% fetal bovine serum (FBS) (164210-50, Pricella, China) and 1% penicillin - streptomycin (PB180120, Pricella, China), and all cells were cultured in a humidified incubator containing 5% CO₂ at 37 °C. In some experiments, the RCC cell lines were treated with GSH at different concentrations. Upon 48 h treatment, the proteins of RCC cell lines were extracted for Western blot assay.

### RNA extraction and RT-qPCR

Total RNA was extracted from frozen specimens and kidney cancer cell lines using TRIzol reagent (9108, Takara, Japan) and cDNA was generated using Evo M-MLV Reverse Transcription Premix Kit (AG11728, Accurate Biology, China). Gene specific cDNAs were amplified by SYBR® Green Pro Taq HS premixed qPCR kit (AG11701, Accurate Biology, China) and the corresponding expression levels were detected by the LightCycler®480 PCR system (Roche). Using GAPDH as the unified control index, the relative expression of genes was analyzed using the 2^-∆∆Ct^ method. The primer sequences were synthesized by Sangon Biotech (Shanghai) Co, Ltd. (China). The primer sequences are listed in Table S1.

### Western blot assay

Total proteins were extracted from tissues or cells using pre-cooled RIPA lysis buffer (P0013B, Beyotime, China) containing protease inhibitors and phosphatase inhibitors. The total protein concentration was then determined using BCA Protein Colorimetric Assay Kit (E-BC-K318-M, Elabscience, China), and the samples were boiled at 100 °C for 5 min. Equal amounts of proteins were separated on a 10% SDS-PAGE gel and transferred to a 0.45 μm PVDF membrane at 165 mA for 90 min. Membranes were closed with 5% skim milk powder and incubated with primary antibody overnight at 4 °C, followed by secondary antibody incubation for 2 h at room temperature. The primary antibodies were CENPT (1:2000, ab86595, Abcam, UK), GCLC (1:1000, abs145984, Absin, China), GCLM (1:2000, ab126704, Abcam, UK), Flag-tag (1:5000, ab205606, Abcam, UK), Myc-tag (1:1000, 2276S, CST, USA), GSH (1:1000, MA1-7620, Invitrogen, USA), ATF2 (1:1000, R380691, Zenbio, China) and GAPDH (1:5000, E-AB-40337, Elabscience, China). The corresponding secondary antibodies (1:10000, E-AB-1003/E-AB-1122, Elabscience, China) were incubated at room temperature for 1 hour.

### Lentivirus infection

Cell transfection targeted knockdown of CENPT using three different lentiviral shRNA oligonucleotide sequences synthesized by OBiO Technology (Shanghai, China). The two most efficient sequences were selected for subsequent analysis. Overexpression recombinant lentivirus was prepared using pSLenti-EF1EGFP-P2A-PuroCMV-MCS-3XFLAG-WPRE lentiviral plasmid carrying green fluorescent protein (GFP) and puromycin resistance gene. The lentivirus CMV-MCS-EF1a-copGFP-PGK-Blast overexpressing GCLC resistant to blasticidin was purchased from Nanjing Zebrafish Biotechnology Co. (China). A 24-well plate was inoculated with 1 × 10^4^ cells per well infected with lentivirus. 48–72 h later, the expression efficiency of fluorescence was initially observed by fluorescence microscopy and 2 times with 5 μg/ml puromycin to screen RCC cell lines for stable expression.

### CCK8 assay

The transfected RCC cells were inoculated in 96-well plates and incubated for 12 h at a concentration of 1 × 10 ^3^ /well with 100 µl of medium. CCK-8 kit (HY-K0301, MCE, USA, 10 µl/well) was added at 24 h, 48 h, 72 h and 96 h post-transfection under dark conditions, respectively. Incubation was performed for 2 h. The absorbance values of each well were detected at 450 nm using an enzyme marker and recorded.

### Colony formation assay

Stable transfer strain RCC cells were inoculated in 6-well plates with 1000 cells per well and incubated for 1 week. The medium was aspirated and fixed with 4% paraformaldehyde for 30 min, followed by staining with 0.1% crystal violet for 20 min. The cells were gently rinsed 2 times with PBS, air-dried, photographed and evaluated by counting with ImageJ software.

### Cell proliferation evaluation

Cell proliferation assays were performed using the BeyoClick™ EdU Cell Proliferation Kit with Alexa Fluor 488 (C0071S, Beyotime, China) and Alexa Fluor 555 (C0075S, Beyotime, China), following the manufacturer’s instructions. Stably transfected cells were incubated with 10 µM EdU for 2 h at 37 °C. Cells were then fixed with 3% paraformaldehyde for 15 min at room temperature and permeabilized with Immunostaining Permeabilization Solution (P0097, Beyotime, China) at room temperature. The fixative was removed and the cells were washed with Immunostaining Closure Solution (P0102, Beyotime, China). Subsequently, cells were incubated in Click additive solution and protected from light and stained with Hoechst. Photographs were then taken under a fluorescent microscope. Statistical analysis was performed by calculating the proportion of EdU-adulterated cells to the total number.

### Wound healing assay

Cells were inoculated in a 6-well plate, scored with a 10 µl pipette tip and washed with PBS to remove unadhered cell debris. Subsequently, photographs were taken at the appropriate time under an inverted fiberscope and the area occupied by cell migration was estimated using ImageJ.

### Transwell assay

For transwell migration and invasion assays, starved RCC cells were suspended in 200 µl of serum-free medium (3 × 10^4^/well) and inoculated into the upper chamber (BD353097, Corning, USA) with (invasion assay) or without (transwell migration assay) Matrigengel (0827045, ABW Bio, China). In the lower chamber, 500 µl of complete medium containing 10% fetal bovine serum was added to the lower chamber. After incubation at 37 °C for 24 h, the cells were fixed with 4% paraformaldehyde for 20 min and stained with 0.1% crystal violet for 15 min. Finally, the stained cells were finally counted under a microscope with three randomly selected fields of view from each chamber.

### Confocal immunofluorescent microscopy

We seeded well-conditioned RCC cells into confocal culture dishes and fixed them with 4% paraformaldehyde at room temperature. After washing with PBS three times, we permeabilized the cells with 0.5% Triton X-100 (P0096, Beyotime, China) at room temperature for 10 minutes. During antibody staining, we used anti-CENPT antibody (1:200, DF2319, Affinity, China) and anti-β-tubulin antibody (1:200, F0167, Selleck, USA), incubating them overnight at 4 °C. The cells were then incubated with fluorescently labeled secondary antibodies at room temperature for 1 hour, followed by three washes with PBS. We incubated the cells with 4′,6-diamidino-2-phenylindole (DAPI) for 5 minutes at room temperature and finally imaged them under a confocal microscope using excitation at 488, 546, and/or 633 nm.

### Co‑Immunoprecipitation (Co‑IP) and mass spectrometry (MS) analyses

Co - IP assays were carried out using stably transfected cells to analyze protein - protein interactions. Antibodies were directly immobilized on an agarose matrix with The Thermo Scientific™ Pierce™ Co - Immunoprecipitation (Co-IP) Kit (26149, Thermo Scientific™, USA). The bait and prey protein mixture was incubated overnight at 4 °C. After the incubation, the eluted and collected immunoprecipitated protein complexes were handed over to Sangon Biotech (Shanghai) Co., Ltd. (China), and MS was used to detect the interacting proteins. The bound proteins were separated by sodium dodecyl sulfate - polyacrylamide gel electrophoresis (SDS - PAGE). When performing Western blot for GSH, a non - reducing sample buffer (such as one without dithiothreitol (DTT) or β - mercaptoethanol) was used to maintain the activity of GSH.

### GST Pull-down

We performed pull-down experiments to determine the direct interactions between proteins. First, we constructed expression vectors of the target proteins with His tags and expressed them in Escherichia coli. The His-tagged fusion proteins were purified to serve as the “bait” proteins. Then, the purified “bait” proteins were immobilized on Ni^2 + -NTA agarose beads. Next, cell lysates containing potential interacting proteins (the “prey” proteins) were added to the beads bound with the “bait” proteins and incubated under suitable conditions to promote interactions between the proteins. After incubation, we washed the beads multiple times to remove non-specifically bound proteins. Finally, SDS-PAGE and Western blot analyses were used to detect the presence of “prey” proteins bound to the beads, thereby verifying the direct interactions between the target proteins.

### Chromatin Immunoprecipitation Assay (ChIP)

ChIP was performed according to the manufacturer’s protocol using the ChIP detection kit (P2078, Beyotime, China). Briefly, RCC cells were treated with 1% formaldehyde for 10 minutes to cross-link DNA and proteins. The cell lysate was sonicated to generate chromatin fragments of 200–300 bp. Immunoprecipitation was then conducted using an anti-ATF2 antibody or IgG as a control. The precipitated chromatin DNA fragments were recovered and analyzed by qRT-PCR. The primer sequences used for ChIP-qPCR detection were listed in Table S2.

### Dual-luciferase reporter assay

The wild-type (WT) and mutant (MUT) sequences of CENPT were synthesized by OBiO Technology (Shanghai, China). The constructed wild-type and mutant plasmids were co-transfected with the ATF2 expression vector (or control vector) into 293 T cells. Lipo8000 (C0533, Beyotime, China) reagent was used for transfection following its operating instructions. The luciferase activity was measured using the Dual-Luciferase Reporter Assay System (RG088S, Beyotime, China). As per the manufacturer’s instructions, a multimode microplate reader was used to measure the luciferase activity.

### Plasmid transfection

Human embryonic kidney HEK293T cells (obtained from Gong Kan lab, Institute of Urology, Peking University., and authenticated by ATCC) were maintained in DMEM medium supplemented with 10% FBS at 37 °C and 5% CO_2_. Plasmid transfection was performed using Lipofectamine™ 8000 reagent (C0533, Beyotime, China) and OPTI-MEM medium (A4124801, Gibco, USA) according to the manufacturer’s protocol. Plasmid pcDNA3-Flag-CENPT and flag-tagged truncator CENPT 334-561aa, 168-333aa, 1-167aa; plasmid pcDNA3-myc-GCLC and myc-tagged truncator GCLC 425-637aa, 213-424aa, 1-212aa, as well as negative control plasmids were all purchased from Nanjing Zebrafish Biotechnology Co.

### Measurements of GSH levels

Cells were scraped off with a cell scraper and collected by adding PBS. For fresh animal tumor samples, we weighed and ultrasonicated homogenized, centrifuged and the supernatant was taken; for nude mice, blood was taken and then rested for 30 min, centrifuged and the serum was taken. GSH content was then determined using a commercially available Total Glutathione (T-GSH) Colorimetric Assay Kit (E-BC-K097-M, Elabscience, China) according to the manufacturer’s protocol.

### Determination of γ-Glutamylcysteine synthetase activity

Stably expressing RCC cells (>5 × 10^6^) were fully lysed on ice using ultrasound. Then, a γ-glutamylcysteine synthetase (GCL) activity assay kit (BA1032, Saint-Bio, China) was used to assess GCL activity via absorbance readings according to the manufacturer’s instructions. GCL activity was normalized to the relative cell count.

### MG132 and Chloroquine treatment

MG132 is an inhibitor of the proteasomal protein degradation pathway, while chloroquine is an inhibitor of the lysosomal pathway. The proteasome inhibitor MG132 (T12628, TargetMol, USA, final concentration of 20 μM), or chloroquine (T8689, TargetMol, USA, final concentration of 40 μM), was added to cells stably expressing CENPT and/or GCLC. The cells were collected for protein extraction and Western blot analysis.

### Cellular ROS and lipid peroxidation detection

In accordance with the manufacturer’s instructions, intracellular ROS generation was assessed using the oxidation-sensitive fluorescent probe DCFH-DA (S0033S, Beyotime, China). In brief, after the specified treatment, cells were incubated in a 37 °C incubator for 30 minutes with culture medium containing 10 µM DCFH-DA. The fluorescence intensity was detected using laser confocal microscopy. The green fluorescence intensity positively correlated with the level of reactive oxygen species. Simultaneously, we also measured the fluorescence intensity using a flow cytometer (analyzed with FlowJo 7.6.1).

For lipid peroxidation detection, we added C11 - BODIPY 581/591 (D3861, Invitrogen, USA) to the culture medium to a final concentration of 1.5 μM. The cells were then incubated at 37 °C for 30 minutes. After the incubation, we washed the cells twice with PBS to remove any unbound dye. Subsequently, we used laser confocal microscopy to detect the fluorescence. The C11-BODIPY 581/591 dye has a distinct property: in its non - peroxidized state, it emits red fluorescence, while in the peroxidized state, it emits green fluorescence. Thus, the ratio of green to red fluorescence intensities corresponds to the level of lipid peroxidation. Specifically, a higher green/red fluorescence ratio indicates a higher level of lipid peroxidation.

### Transmission Electron Microscopy (TEM)

Cells were cultured in a 10 cm dish, and the medium was discarded. The cells were gently washed twice with pre-cooled PBS at 4 °C. Subsequently, a cell scraper was used to quickly remove the cells, which were then transferred entirely into an EP tube for centrifugation at 1000 rpm at low temperature for 5 minutes. The supernatant was then removed, and a sufficient amount of pre-cooled 2.5% glutaraldehyde was immediately added for fixation at 4 °C for 24 hours. After rinsing with 0.1 M phosphate buffer, the cells were fixed with 1% OsO4. The cell pellet was dehydrated and embedded in resin to form 60–80 nm cell sections. After staining with uranyl acetate and lead nitrate, the cells were examined using Hitachi HT7800 TEM.

### Xenograft models

Six - week - old female BALB/c - nude mice were purchased from Jinan Pengyue Experimental Animal Breeding Co (Jinan, China). All procedures were approved by the Ethics Committee of the Medical Animal Laboratory of the Affiliated Hospital of Qingdao University (No: AHQU-MAL20230512). In this animal study, no randomization method was used to allocate the mice to experimental groups. The mice were simply used for establishing the xenograft tumor model in a sequential manner. A xenograft tumor model was established by subcutaneously injecting 5 × 10⁶ tumor cells into the right side of nude mice. The length and width of the tumors were measured every three days. The tumor volume was calculated by the formula: TV (mm³) = length × width² × 0.5. At the end of the experiment, the mice were euthanized and the tumor tissues were weighed. Tumor size was measured and glutathione content was assayed. In this study, the exclusion criteria for the experimental mice were as follows: Mice were excluded from the analysis if they showed severe non - tumor - related illnesses, abnormal weight loss during the experiment, or no obvious tumor growth within 7 days after tumor implantation. These criteria were pre - established before the start of the experiment to ensure the reliability and accuracy of the experimental results.

### Statistical analysis

Prior to any comparisons, we employed Levene’s test to assess the homogeneity of variance. For two - group data comparisons with homogeneous variances, we used two - tailed Student’s t - test via GraphPad Prism 8.0; for heterogeneous variances, Welch’s t - test was applied. For more than two - group data, when variances were equal, one - way ANOVA was conducted using GraphPad Prism 7.0 followed by post - hoc Tukey’s HSD test if significant, and for unequal variances, the Kruskal - Wallis test was used. Survival curve analysis was done by the Kaplan - Meier method with log - rank tests for comparison. CCK8 assays for cell proliferation used two - way ANOVA after checking relevant assumptions including variance homogeneity. All *p* values < 0.05 were considered significant. In in vivo studies, *n* is the number of individual mice, and in in vitro cell culture studies, n represents independent experiments.

## Results

### CENPT correlated with prognosis in RCC

**Fig. 1:**
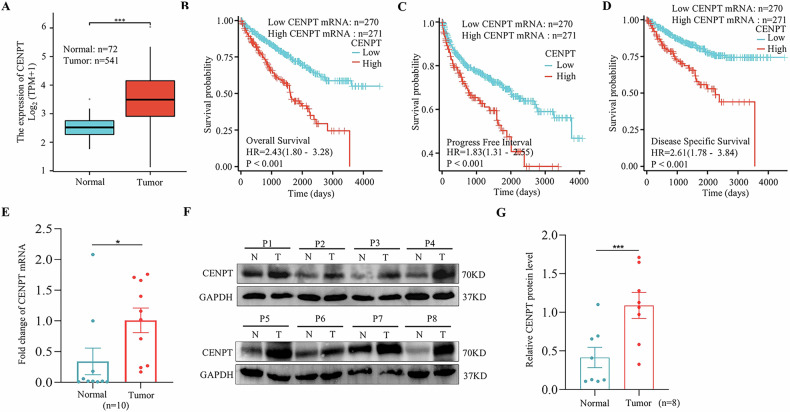
The expression of CENPT was correlated with the prognosis of RCC. **A** Relative expression of CENPT in RCC samples from the TCGA database; The prognostic curves of different CENPT expression levels in RCC samples were analyzed. Kaplan-Meier analysis of the correlation between CENPT levels and overall survival (**B**), progress free interval (**C**) and disease specific survival (**D**) in RCC samples; **E** qRT-PCR was used to analyze the mRNA expression level of CENPT in tumor tissues and paratumor samples from RCC patients. **F**, **G** Western blot analysis of the protein expression level of CENPT in tumor tissues and paratumor samples from RCC patients. Data are mean ± SEM. The *n* number represents n biologically independent patient samples in each group. Exact *n* values are marked in the images. Statistical significance was determined by Student’s *t*-test (**A**, **E**), Log-rank test (**B**–**D**) and t-test (two tail) (**G**). Compared with the indicated group, *p < 0.05, ***p < 0.001.

To identify the roles of centromere and kinetochore genes in RCC, we investigated the potential cancer prognosis of 31 centromere and kinetochore protein genes using RNA expression profiles from TCGA database with the R programming language. Our findings indicated that CENPT’s hazard ratio (HR) ranks among the top five, and its role in cancer has not been previously elucidated (Table S3). And the mRNA expression of CENPT was shown to be remarkably elevated in RCC samples (Fig. 1A). Then, survival analysis showed that CENPT overexpression in RCC samples was significantly associated with poorer overall survival, progress free interval and disease specific survival (Fig. 1B–D). In addition, we also collected 10 pairs of kidney cancer and paraneoplastic tissues in our center and determined the expression level of CENPT in these patient tissue samples. Consistent with the TCGA RNA expression data analysis results (Fig. 1A), the expression of CENPT is significantly upregulated in kidney cancer tissues (Fig. 1E, *p* < 0.05). At the protein level, CENPT expression was relatively higher in tumor tissues of 8 RCC patients than in matched paraneoplastic tissues (Fig. 1F, G, *p* < 0.001). Hence, these results suggest that CENPT may play a role in the tumorigenesis and progression of RCC.

### CENPT promoted the development of RCC cells by inhibiting ferroptosis

**Fig. 2:**
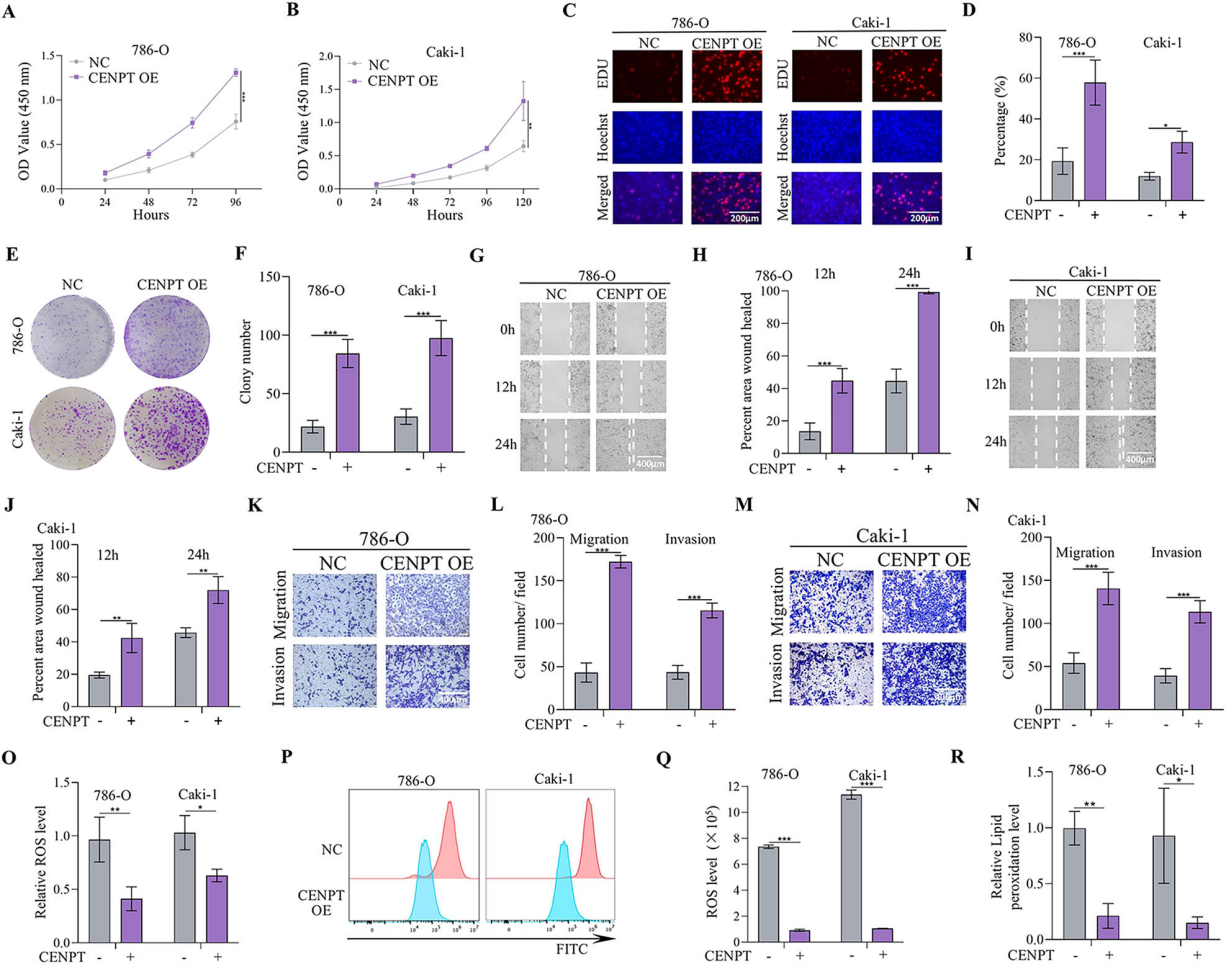
Overexpression of CENPT promoted the progression of RCC by inhibiting ferroptosis. **A–D** CCK-8 and EdU immunofluorescence staining of 786-O and Caki-1 cells transfected with indicated overexpression plasmids. **E**, **F** Colony formation assay analyzing cell proliferation in different cells. **G**–**J** Wound healing assays were performed in different cell lines overexpressing CENPT and control cell lines, respectively, and quantitatively analyzed. **K**–**N** Transwell assay assessing cell migration and invasion abilities in 786-O cells and Caki-1 cells. **O**–**R** Intracellular ROS and lipid peroxidation levels in different RCC cells. Data (means ± SEM, *n* = 3) were representative of three separate experiments with similar results. Compared with the indicated group, *p < 0.05, **p < 0.01, ***p < 0.001.

Next, the expression levels of CENPT in RCC cell lines were assessed. Results indicated that CENPT expression was higher in A498 and ACHN cell lines compared to OSRC-2, 786-O, and Caki-1 cell lines (fig S1A, B). In contrast, the expression of CENPT was lower in 786-O and Caki-1 cell lines compared to other RCC cells (fig S1A, B). To evaluate the role of CENPT in regulating RCC phenotypes, short hairpin RNA (shRNA) lentiviruses (sh-Control, sh-CENPT-1/2/3) and overexpression lentiviruses were generated. Subsequently, overexpression lentiviruses targeting CENPT were stably transfected into 786-O and Caki-1 cell lines (the overexpression efficiency of the transfected RCC cells was shown in fig S1C–E), while CENPT shRNA lentiviruses were stably transfected into A498 and ACHN cells (the target sequences of CENPT shRNA were shown in Table S4, and the knockdown efficiency of the transfected RCC cells was shown in fig S1F–I). Functional assays were conducted in vitro to assess CENPT’s role in RCC cell lines. CCK8 and EdU assays revealed that overexpression of CENPT promoted the proliferation of 786-O and Caki-1 cells (Fig. 2A–D). Colony formation experiments further demonstrated that CENPT overexpression significantly enhanced cell proliferation (Fig. 2E, F), indicating a promotion of tumor growth in vitro. Additionally, wound healing and transwell assays showed that CENPT overexpression facilitated the migration and invasion of RCC cells (Fig. 2G–N). Interestingly, overexpression of CENPT significantly reduced ROS and lipid peroxidation levels (Fig. 2O–R).

**Fig. 3:**
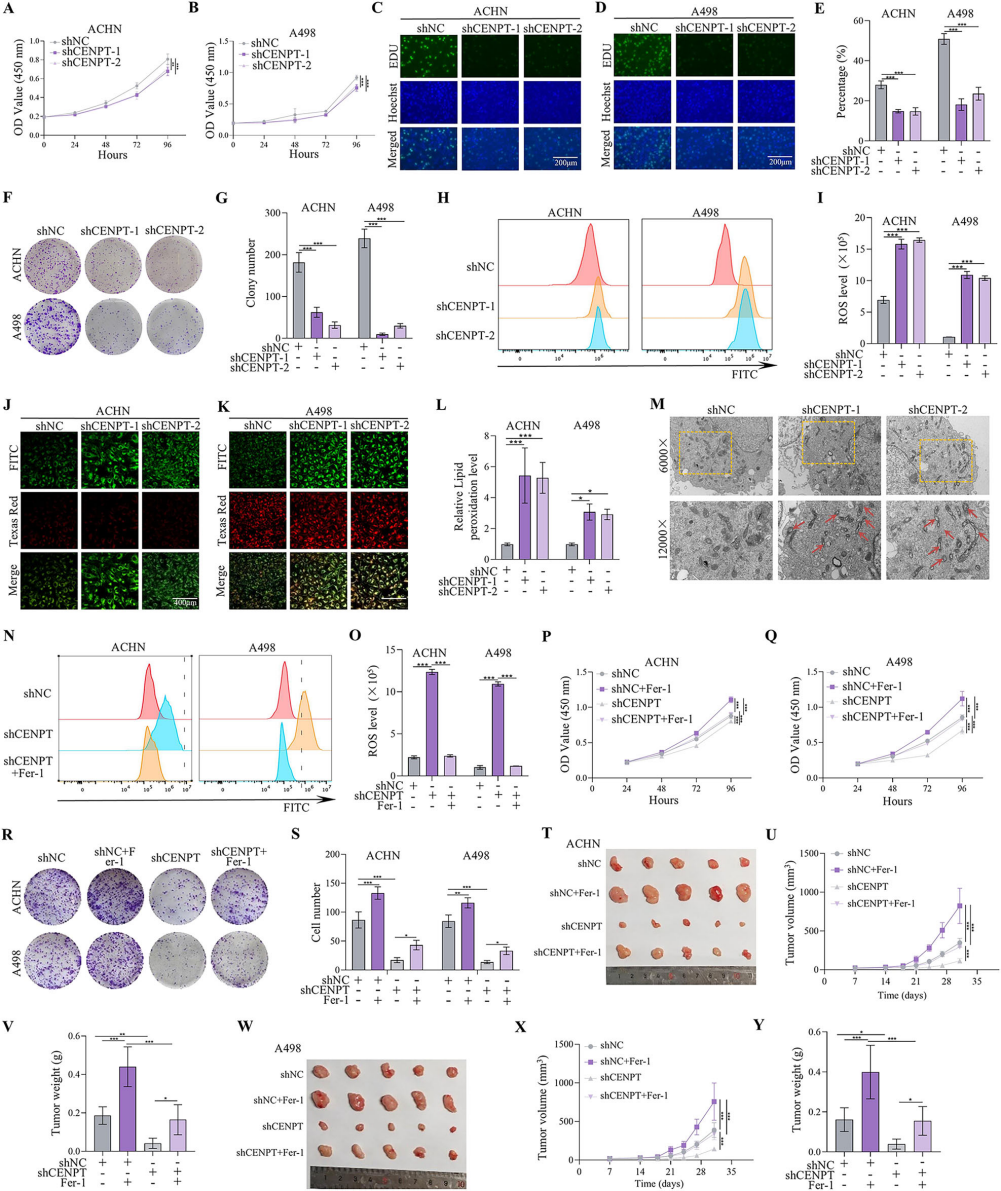
Ferroptosis inhibitor Fer-1 inhibited shCENPT-mediated RCC ferroptosis in vitro and in vivo. **A**, **B** CCK-8 assay analyzing cell proliferation in different cells. **C**–**E** EdU immunofluorescence staining of ACHN and A498 cells transfected with indicated plasmids (20x). **F**, **G** Colony formation assay assessing cell proliferative ability in different cells. **H** Intracellular ROS levels in A498 and ACHN cells transfected with indicated shRNA lentiviruses. **J**–**L** Detection of lipid peroxidation levels of RCC cells transfected with shNC, shCENPT-1, and shCENPT-2. The green fluorescence (FITC channel) corresponds to peroxidized lipids, while the red fluorescence (Texas Red channel) corresponds to non - peroxidized lipids. **M** Electron micrographs in ACHN cells with CENPT knocked down. The red arrows indicate that the mitochondrial cristae have increased electron density and are visibly condensed and reduced in size. **N**, **O** The cellular ROS level was analyzed by a flow cytometer. **P**–**S** The CCK-8 and colony formation assay in CENPT stably knockdown RCC cells with or without Fer-1 treatment. **T**, **Y** The in vivo effect of Fer-1 treatment on shCENPT-mediated anti-RCC tumor therapy. **T**, **W** Representative images of subcutaneous tumors derived from a xenograft model using different RCC cells. **U**, **X** Tumor growth curve of different groups. **V**, **Y** The tumor weights of RCCs stably transfected with NC or shCENPT with or without Fer-1 treatment. Data are given as mean ± SEM (in vitro assays, *n* = 3; in vivo assays, *n* = 5). Compared with the indicated group, *p < 0.05, **p < 0.01, ***p < 0.001.

Conversely, after constructing two shRNA targeting CENPT using lentiviral vectors, CCK-8 and EdU assays indicated a decrease in the proliferation capacity of RCC cells following CENPT knockdown (Fig. 3A–E). Colony formation assay results showed that silencing CENPT significantly downregulated RCC cell proliferation (Fig. 3F, G). Moreover, RCC cell migration and invasion abilities were hindered by CENPT knockdown (fig S2A–H). Furthermore, the dual transfection of lentiviral vectors for knockdown and overexpression of CENPT validated its oncogenic role in RCC cell lines (fig S3A–G). In summary, these findings suggest that CENPT promotes the proliferation, migration, and invasion of RCC cells.

We subsequently established a nude mouse model to gain deeper insights into the in vivo role of CENPT in RCC progression. CENPT stably overexpressed Caki-1/786-O cells (CENPT OE) and corresponding control Caki-1/786-Ocells (NC) were generated and subcutaneously injected into the right axils of nude mice. The findings demonstrated that CENPT overexpression significantly promoted tumor growth, resulting in notably larger tumor volumes and weights compared to the control group (fig S3H–J/S3L–N). Collectively, these data unequivocally confirmed the pro-carcinogenic role of CENPT in the progression of RCC. To further substantiate the impact of CENPT on the survival of tumor-bearing mice, we injected CENPT stably overexpressed Caki-1/786-O cells or corresponding controls into the subcutaneous right forelimbs of nude mice. As anticipated, Kaplan-Meier analysis unveiled that the survival period of the CENPT overexpressing group of mice was shorter than that of the vector-control mice (fig S3K/O). In summary, these results strongly indicate that CENPT markedly promotes the progression of RCC and shortens the survival period of tumor-bearing mice.

Notably, the abnormal expression of centromere genes often led to chromosomal instability (CIN), thereby promoting tumorigenesis and progression [25, 26]. As a component of the centromere, CENPT might have influenced normal chromosome segregation, resulting in the occurrence of CIN [27, 28]. To investigate whether CENPT expression induced chromatin division defects, we used DAPI staining to visualize the nuclei of shNC- and shCENPT-treated cells. In RCC cells subjected to different transfections, we observed round micronuclei, and there was no significant difference in the number of multinucleated cells between the two groups (fig S4A, B). The presence of cells with micronuclei indicated abnormal chromosome segregation events during mitosis, suggesting that shCENPT did not enhance chromosome segregation defects in RCC. Considering that ROS accumulation and lipid peroxidation are key factors triggering ferroptosis, we hypothesized a relationship between ferroptosis and CENPT. Indeed, we observed that shCENPT markedly promoted ROS and lipid peroxidation levels (Fig. 3H–L and fig S4C, D), thereby inducing ferroptosis of RCC cells. In addition, ultrastructural analysis showed mitochondrial shrinkage, cristae reduction or loss, and increased membrane density in RCC after CENPT knockdown, which are ferroptosis-specific morphologies (Fig. 3M). Ferrostatin-1 (Fer-1) is a specific inhibitor of ferroptosis used to assess the role of ferroptosis in regulating tumor development [29]. After treatment with Fer-1 (5 μM), we observed an increase in the expression levels of CENPT in RCC cell lines (fig S5A–C). In addition, we performed DCFH-DA staining and detected by flow cytometry, and found that the ROS levels produced by the cells after Fer-1 treatment were decreased (Fig. 3N, O). Correspondingly, in the presence of Fer-1, the inhibitory effect of shCENPT on the growth of RCC cells was reversed (Fig. 3P–S and fig S6A–H). Furthermore, the in vivo results showed that Fer-1 (One week after subcutaneous tumor implantation, Fer-1 (10 mg/kg) was injected peri-tumorally every other day until the animals died.) had the same impact on the shCENPT**-**mediated ferroptosis (Fig. 3T–Y). Therefore, CENPT prevented RCC cells against ferroptosis.

### CENPT reversed the inhibitory effect of ferroptosis activators on RCC growth

Ferroptosis is a form of iron-dependent programmed cell death caused by the lethal accumulation of lipid peroxides in cell membranes, mechanistically and morphologically distinct from other forms of cell death such as apoptosis and necroptosis [30, 31]. Since ferroptosis is related to ROS and GSH regulation, as expected, we found that Fer-1 treatment significantly restored cell viability in CENPT knockdown cells. Therefore, we sought to determine whether CENPT overexpression inhibited ferroptosis in RCC cells. Erastin inhibited system Xc- and prevented the import of cystine, thereby reducing GSH levels and promoting lipid peroxidation, which in turn promoted ferroptosis [32]. Accordingly, in cells overexpressing CENPT, we performed erastin treatment and observed that overexpression of CENPT reversed the pro-ferroptotic effect of erastin on RCC and reversed erastin’s inhibitory effect on RCC growth (fig S7A–L).

### CENPT was the binding partner of GCLC

**Fig. 4:**
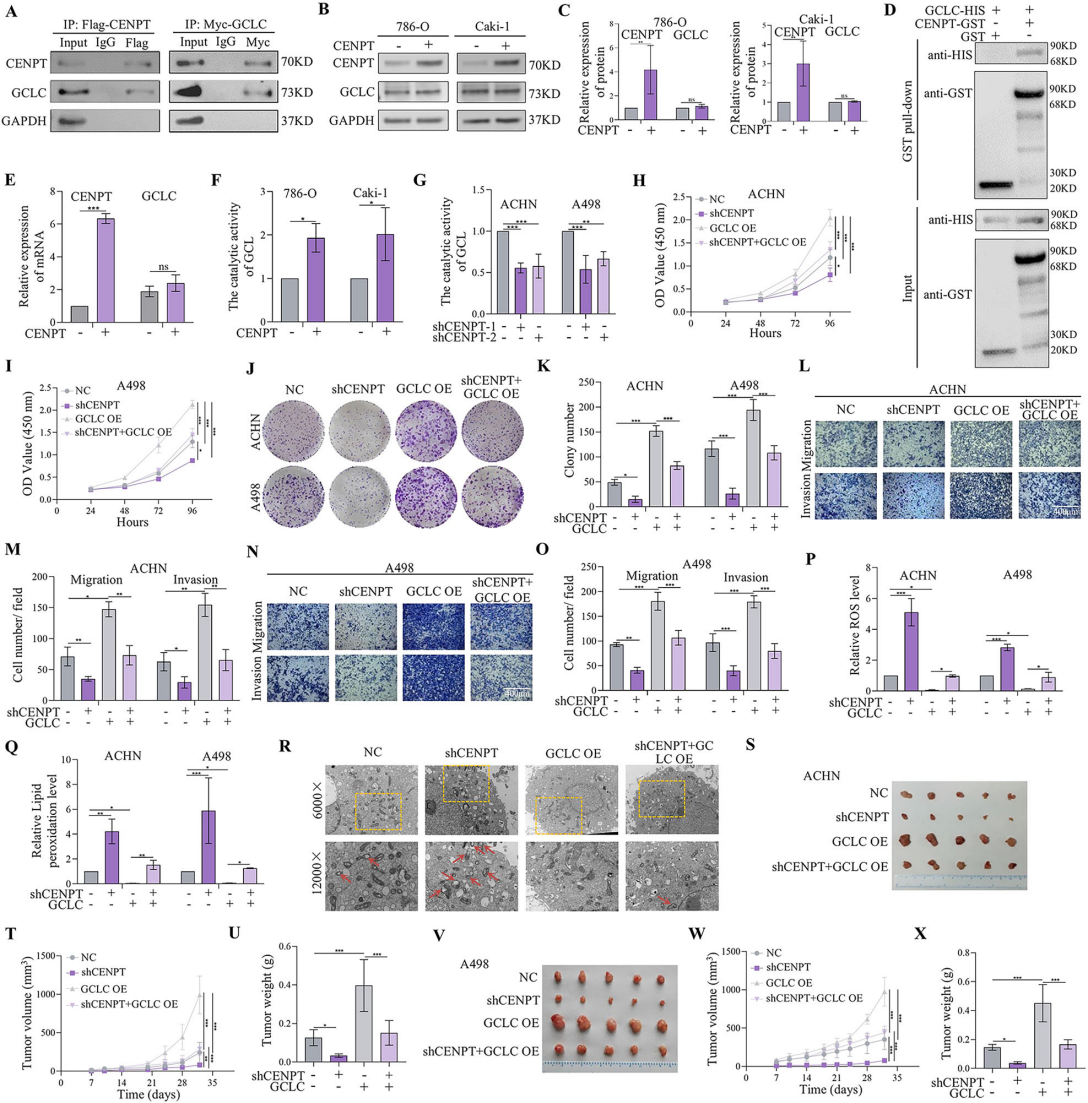
CENPT promoted RCC tumor proliferation and metastasis by increasing GSH synthesis in vitro and in vivo. **A** The interaction between CENPT and GCLC proteins was detected by co-immunoprecipitation assay. **B**, **C** Protein levels of GCLC in RCC cells after CENPT overexpression. **D** CENPT/GCLC interaction by GST-affinity pull-down assay. GST-CENPT was captured by glutathione-resin to probe the presence of HIS-GCLC with western blotting. **E** mRNA levels of GCLC in RCC cells after CENPT overexpression. **F**, **G** The catalytic activity of GCL in RCC cells after CENPT overexpression or knockdown. **H**–**K** The proliferation of RCC cells was detected by CCK8 and colony formation assay after co-transfection of shCENPT with GCLC overexpression. **L**–**O** Transwell assay suggested that GCLC overexpression reversed the inhibitory effect of knockdown of CENPT on migration and invasion. **P**, **Q** Intracellular ROS and lipid peroxidation level in different RCC cells. **R** Electron microscopy of RCCC cells after GCLC interaction with CENPT. Red arrows indicate damage to the mitochondrial membrane, increased or decreased membrane density, etc. **R**–**X** The in vivo effect of GCLC overexpression on shCENPT-mediated anti-RCC tumor growth. **S**, **V** Gross image of subcutaneous tumors. **T**, **W** Tumor growth curve of different groups. **U**, **X** The tumor weights of different groups. Data are given as mean ± SEM (in vitro assays, *n* = 3; in vivo assays, *n* = 5). Compared with the indicated group, *p < 0.05, **p < 0.01, ***p < 0.001.

Having established that CENPT promoted the progression of RCC by inhibiting ferroptosis. We then investigated the underlying mechanism that CENPT increased the development of tumor. To identify the potential CENPT interactors, we performed co-immunoprecipitation and mass spectroscopy experiments. As a result, the enriched CENPT-associated protein complexes were analyzed by mass spectrometry and candidate proteins were detected compared to IgG control samples. Further analysis of these proteins showed a high enrichment with the fatty acid metabolism and ferroptosis pathway (fig S8A). Then, we further validated whether the key nuclear and cytoplasmic proteins of the above-mentioned three pathways interacts with CENPT by using WB. In brief, we transfected FLAG-tagged CENPT constructs and MYC-tagged GCLC into 293 T cells and tested the interaction between CENPT and GCLC using Co-IP and WB (Fig. 4A). Similarly, we detected a significant interaction between endogenous CENPT and GCLC in RCC cells (fig S8B, C). These observations indicated that the interaction between CENPT and GCLC might induce the malignant progression of RCC. We found that overexpression of CENPT did not significantly alter the protein levels of GCLC (Fig. 4B, C). As expected, this direct interaction was confirmed by an in vitro GST pull-down assay (Fig. 4D). Furthermore, overexpression of CENPT did not significantly change the mRNA levels of GCLC (Fig. 4E), but it increased the catalytic activity of GCL (Fig. 4F). Additionally, we found that shCENPT significantly reduced the catalytic activity of GCL (Fig. 4G). Then, to further test this hypothesis, we generated CENPT stably knockdown and GCLC stably overexpressing ACHN/A498 cell lines by using lentivirus. Therefore, we further explored whether the proteasome inhibitor or autophagy inhibitor interfered with the effect of CENPT on the expression level of the GCLC. Interestingly, neither the proteasome inhibitor MG132 nor the autophagy inhibitor chloroquine had any effect (fig S8D–K) on the GCLC expression. These findings showed that CENPT increased the catalytic activity of GCLC but had no effect on the GCLC protein’s stability or expression.

Subsequently, CCK8 and colony formation experiments validated that the interaction between GCLC and CENPT was essential for promoting the proliferation of RCC (Fig. 4H–K). In addition, results of wound healing assays in ACHN and A498 cells showed that overexpression of GCLC significantly restored the migratory capability of RCC cancer cells against CENPT shRNA (fig S8L–O), and transwell assay also showed that overexpression of GCLC markedly rescued the inhibitory effects of CENPT shRNA on RCC cancer cell migration and invasion (Fig. 4L–O). Simultaneously, overexpression of GCLC also suppressed the elevation of ROS and lipid peroxidation levels induced by shCENPT (Fig. 4P, Q and fig S8P, Q). In addition, after CENPT knockdown, RCC showed mitochondrial contraction and increased membrane density, and this ferroptosis-specific morphology was reversed by GCLC (Fig. 4R), consequently inhibiting shCENPT-induced ferroptosis. These findings showed that CENPT promoted the progression of RCC by interacting with GCLC and increasing the catalytic activity of GCLC.

Furthermore, to explore the in vivo effect of CENPT-GCLC axis on the progression of RCC, we injected subcutaneously CENPT stably knockdown A498 cells, GCLC stably overexpressing A498 and ACHN cells individually or in combination, and corresponding controls into the axils of nude mice. As shown in Fig. 4S–X, overexpression of GCLC almost completely rescued the inhibitory effects of CENPT silencing on mouse tumor growth. Collectively, GCLC was crucial for CENPT-induced proliferation and metastasis of RCC by inhibiting ferroptosis.

### CENPT Bound to the Amino Acid Region 213 – 424 of GCLC

**Fig. 5:**
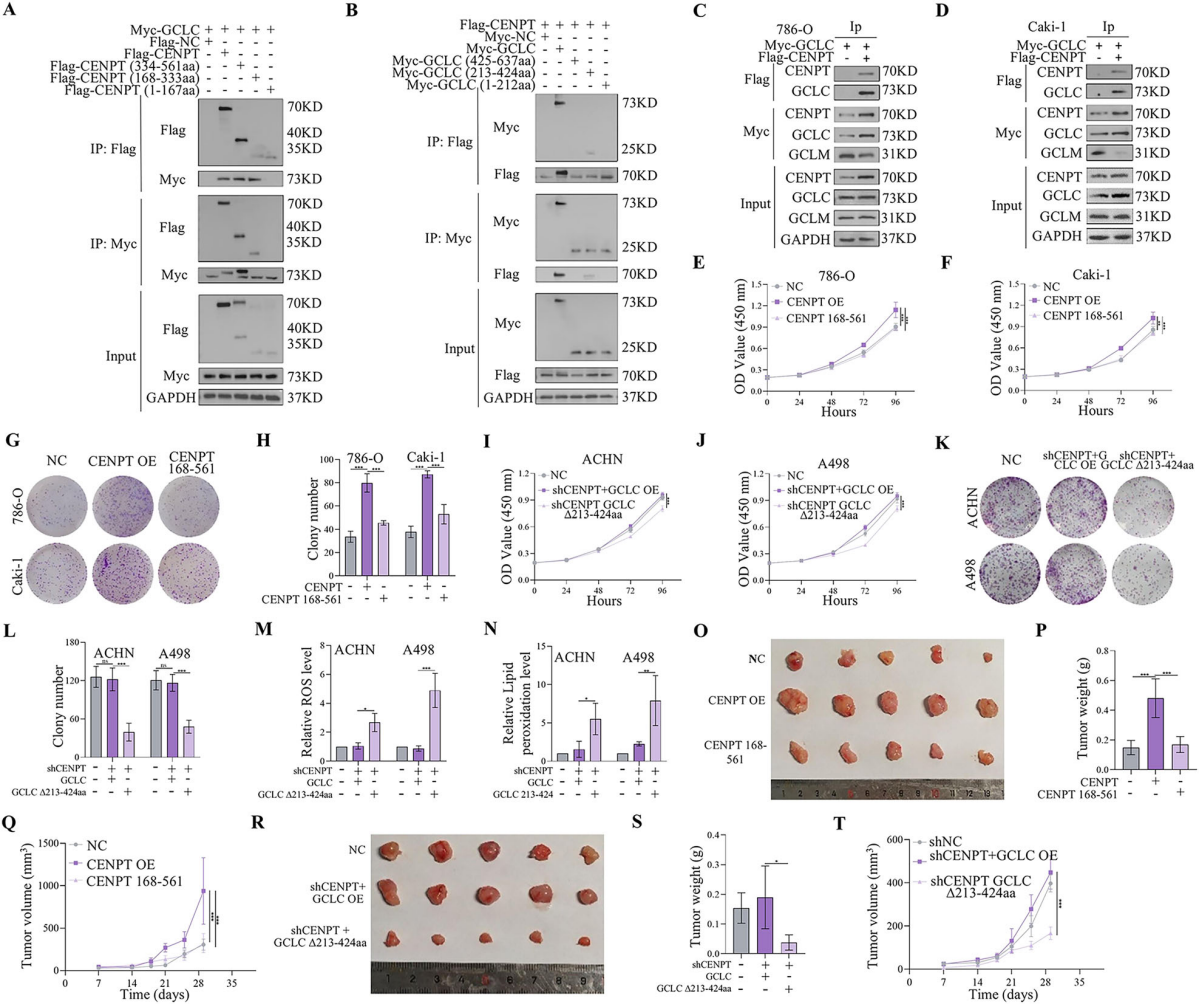
CENPT interacts with GCLC. **A** Immunoprecipitation revealed that GCLC binds to the 168-561 structural domain of CENPT. **B** CENPT directly interacts with the 213-424 structural domain of GCLC. **C**, **D** CENPT directly binds to GCLC competitively with GCLM. **E**–**H** Cell lines stably transfected with CENPT overexpression and truncator were tested for proliferation of RCC cells by CCK8 and clone formation assay. **I**–**L** Proliferation of RCC cells was detected by CCK8 and clone formation assays after co-transfection of shCENPT with GCLC overexpression as well as the truncator. **M**, **N** The overexpression of GCLC reversed the promoting effect of shCENPT on intracellular lipid peroxidation and ROS levels, while the GCLC mutant did not exhibit this effect. **O**–**T** The in vivo effect of CENPT and GCLC deletion mutants overexpression on tumor growth. **O**, **R** Gross image of subcutaneous tumors. **P**, **S** The tumor weights of different groups. **Q**, **T** Tumor growth curve of different groups. Data are given as mean ± SEM (in vitro assays, *n* = 3; in vivo assays, *n* = 5). Compared with the indicated groups, *p < 0.05, **p < 0.01, ***p < 0.001.

To further determine the precise binding region of CENPT and GCLC, we enforced the expression of full-length Myc-GCLC and fragmented Flag-CENPT in 293 T cells. Through a Co-IP assay, we found that GCLC bound to the ${\boldsymbol{\triangle }}$N-terminal (amino acids 168–561 or 168–561aa) domain of CENPT (Fig. 5A).

We next investigated which domain of GCLC is required for CENPT binding. Then, we enhanced the expression of the N-terminal (amino acids 1–212), middle fragment (amino acids 213–424) and C-terminal (amino acids 425–637) of Myc-GCLC and full-length CENPT in 293 T cells. Through Co-IP assays, we found that CENPT bond to the middle fragment (amino acids 213-424) of GCLC (Fig. 5B). Altogether, these results demonstrated a direct interaction between CENPT and GCLC.

Our results showed that CENPT bound to the GCLC mutant form carrying deletion of ∆213-424 aa (GCLC ∆213–424aa), which domain was also required for GCLM binding [33]. Hence, to investigate whether the interaction between CENPT and GCLC affect GCLM binding to GCLC, we performed the CO-IP assay. In Fig. 5C, D, it showed that CENPT directly binds to GCLC competitively with GCLM.

To further determine the binding domain of CENPT is crucial for their roles in the development of RCC cells, we determined the proliferative capacity of the full-length CENPT and its regional truncations in different cell lines. Results from CCK-8 and EDU assays indicated that the truncated forms of CENPT 168–333aa, CENPT 334-561aa, and full-length CENPT all promoted the proliferation of RCC, whereas no promoting effect was observed with the truncated form of CENPT 168 – 561aa (fig S9A–E). The results suggest that the promotion of RCC development is mediated by the interaction between the CENPT 1–167 and GCLC.

Furthermore, we enforced the expression of full-length CENPT, fragmented CENPT 168–561, full-length GCLC and GCLC ∆213–424aa in RCC cells (the overexpression efficiency of transfected RCC cells was shown in (fig S9F–I). Notably, overexpression of CENPT 168–561 could not increase the development of RCC, compared to the full-length CENPT overexpression group (Fig. 5E–H and fig S10A–H). Furthermore, overexpression of GCLC ∆213-424aa also could not reverse shCENPT-mediated inhibitory effect on the progression of RCC (Fig. 5I–L and fig S10I–P) and ferroptosis (Fig. 5M, N). Additionally, the in vivo results also showed that overexpression of CENPT 168–561 could not promote RCC tumor growth, compared to the full-length CENPT overexpression group (Fig. 5O–Q). Overexpression of GCLC ∆213–424aa also could not reverse shCENPT-mediated inhibitory effect on the RCC tumor growth (Fig. 5R–T). Therefore, these results indicated that CENPT directly binds to GCLC competitively with GCLM, thereby prevents RCC against ferroptosis.

### CENPT promoted RCC tumor proliferation and metastasis by increasing GSH synthesis

**Fig. 6:**
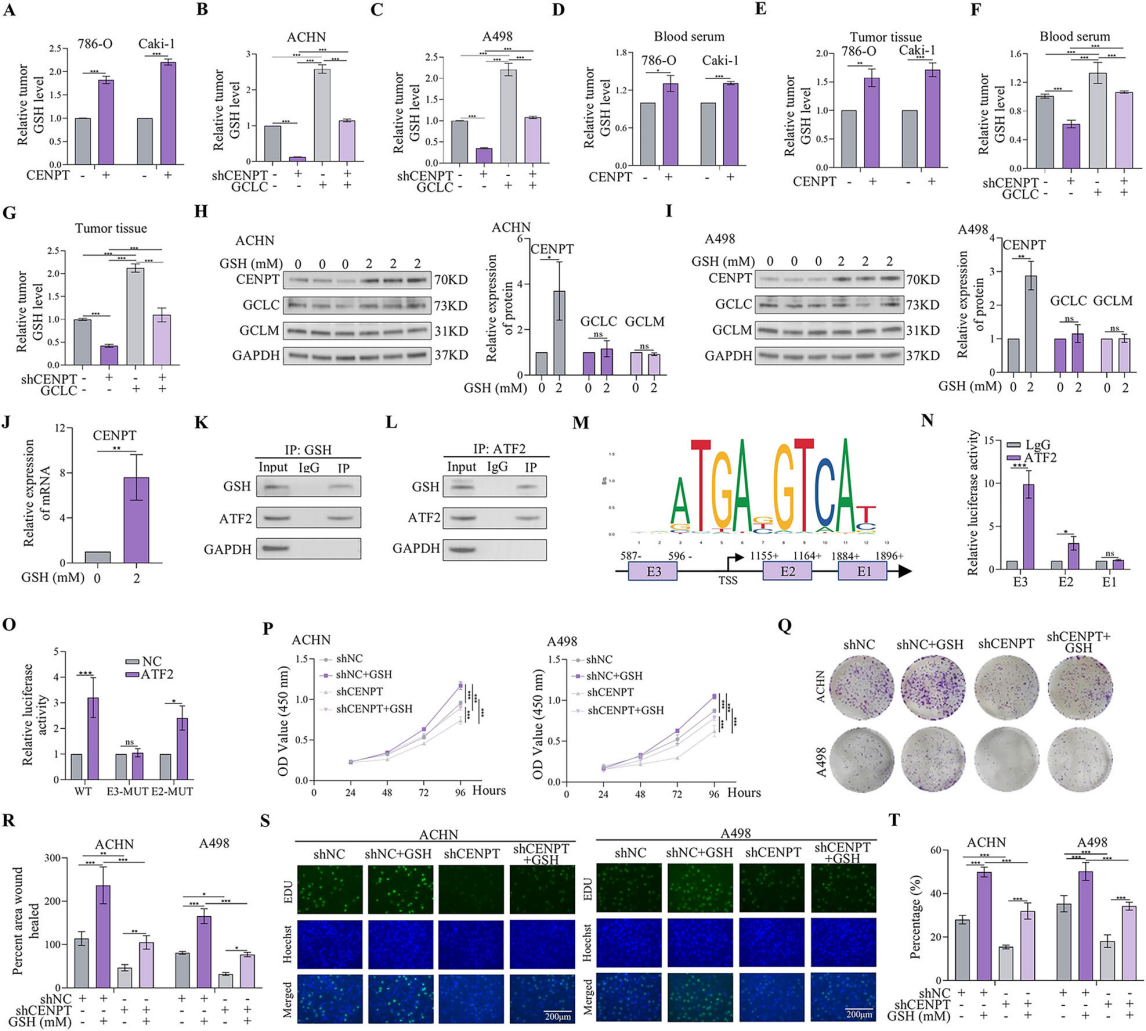
GSH reversed the inhibitory effects of shCENPT on the growth, migration, and invasion of RCC. **A** The level of intracellular GSH content increased after overexpression of CENPT. **B**, **C** Intracellular GSH levels decreased after CENPT inhibition, which was reversed by overexpression of GCLC. **D**–**G** The expression of CENPT affects the expression level of GSH in vivo. **H**, **I** GSH increased protein expression levels of CENPT and GCLC in different cell lines. **J** mRNA Expression of CENPT in RCC cells after GSH treatment. **K**, **L** Co-IP assays demonstrated the direct binding between GSH and ATF2. **M** The predicted binding sites of ATF2 to the promoter of CENPT. **N** ChIP-PCR experiments demonstrated direct binding of ATF2 to the promoter regions of CENPT in RCC cells. **O** The luciferase reporter gene assay was used to determine the ATF2 binding sites on the CENPT promoter region. **P** Following GSH treatment, the CCK-8 assay was employed to determine the growth curve of shCENPT-treated RCC cells. **Q**, **R** Colony formation assays were conducted to assess the proliferative capacity of shCENPT-treated RCC cells under the influence of GSH. **S**, **T** The EdU incorporation assay was utilized to detect the proliferation status of different RCC cells after GSH treatment. Data are given as mean ± SEM (*n* = 3). Compared with the indicated groups, *p < 0.05, **p < 0.01, ***p < 0.001.

Previous studies showed that GCLC directly interacted with GCLM. GCLM is enzymatically inactive but plays an important regulatory function by lowering the Km of GCL for glutamate and raising the Ki value of GSH feedback inhibition [34, 35]. GCLC is the catalytic subunit of GCL, which is the rate-limiting enzyme for GSH synthesis. To further investigate the effect of CENPT and GCLC on the GSH synthesis, we determined the intracellular GSH levels after overexpression of CENPT. As a result, the intracellular GSH levels were increased by the overexpression of CENPT (Fig. 6A). Overexpression of GCLC markedly rescued the inhibitory effects of CENPT shRNA on the GSH synthesis in different RCC cells (Fig. 6B, C).

In addition, overexpression of CENPT had the capacity to increase the GSH levels in mouse serum and tumor tissues (Fig. 6D, E). However, overexpression of GCLC almost completely rescued the inhibitory effects of CENPT silencing on GSH levels in mouse serum and tumor tissues (Fig. 6F, G). Therefore, these results revealed that CENPT promoted RCC tumor progression by increasing GSH synthesis.

### Feedback mechanism of GSH regulating CENPT expression via ATF2

GSH is a nonallosteric feedback inhibitor of GCL, but the binding of GSH to the enzyme competes with glutamate [36]. However, we found that the interaction between CENPT and GCLC increased GSH levels in serum, cells and tissues. Therefore, we hypothesized that GSH might influence the activity of GCL under certain circumstances. To explore this possibility, we measured the expression levels of CENPT and GCLC using Western Blot after GSH stimulation. GSH exhibited a biphasic regulatory effect on CENPT protein expression in RCC cell lines. GSH exerts a biphasic regulatory effect on CENPT protein expression in RCC cell lines. Treatment with GSH significantly enhances CENPT protein levels, with the optimal promoting effect at 2 mM (Fig. 6H, I and fig S11A–D). This dose-dependent pattern suggests a potential therapeutic window for GSH-mediated CENPT modulation. Interestingly, GSH also induced the expression of CENPT at the transcriptional level by activating its promoter (Fig. 6J). These findings suggested that there was a correlation between GSH and the expression of CENPT, possibly influencing the expression level of CENPT through certain mechanisms. Based on current research, Activating Transcription Factor 2 (ATF2) was widely recognized as an important transcription factor involved in regulating various biological processes, including cell proliferation, apoptosis, and stress responses[37, 38]. From transcriptome sequencing results, we hypothesized that ATF2 might regulate the transcription level of CENPT by binding to its promoter region. First, we used immunoprecipitation and Western blotting to evaluate the interaction between GSH and ATF2 (Fig. 6K, L). Then, through database analysis, we predicted the potential binding sites of ATF2 on the CENPT promoter region (Fig. 6M). The E1 binding site was located inside intron 2 (+1896 to +1912 bp), the E2 binding site resided at the junction between exon 1 and intron 1 (+596 to +612 bp), and the E3 binding site was positioned in the promoter region (−1200 to −1184 bp). PCR following ATF2 ChIP demonstrated that the anti-ATF2 antibody was significantly enriched in the E2 and E3 regions (Fig. 6N). To validate the direct activation of the CENPT promoter by ATF2, we constructed luciferase reporter vectors containing different binding sites. Results showed that overexpressing ATF2 in 293 T cells enhanced the luciferase activity of the WT vector by 3.2-fold, whereas E3-MUT site only increased the activity by 1.1-fold (Fig. 6O)—confirming that E3 served as a critical site for ATF2 to regulate CENPT transcription. Further investigations revealed that ATF2 overexpression significantly elevated both CENPT mRNA and protein levels (fig S11E/G), while ATF2 knockdown (sh-ATF2) suppressed CENPT expression in RCC cell lines (fig S11F/H). Additionally, after ATF2 knockdown, CENPT mRNA in sh-ATF2 cells showed no response to GSH treatment, and sh-ATF2 completely blocked the GSH-induced upregulation of CENPT protein (fig S11I–J). These experiments collectively confirmed that ATF2 played a pivotal mediating role in the GSH-induced CENPT expression pathway. These observations explained the positive regulation of CENPT mRNA expression by GSH through the transcription factor ATF2. Next, co-transfection of sh-ATF2 and CENPT overexpression was conducted to validate cell proliferation and progression; results demonstrated that ATF2 knockdown effectively counteracted the promoting effects of CENPT overexpression on these biological behaviors (fig S11K–O). Collectively, these experiments systematically established a robust evidential chain for ATF2’s regulatory role in CENPT expression and its functional impacts, providing substantial experimental support for elucidating the underlying mechanisms by which ATF2 mediates CENPT to influence cellular processes. Furthermore, knockdown of CENPT significantly suppressed the promoting effect of GSH on RCC cell proliferation, migration, and invasion (Fig. 6P–T, fig S11P–W). Therefore, these findings thus demonstrated not only a novel mechanism by which GSH regulated CENPT expression through ATF2, but also solidified ATF2’s central mediating role in the “GSH-CENPT” functional axis.

## Discussion

Centromere and kinetochore gene misexpression played a vital role in tumor progression [8]. In this study, we investigated the potential cancer prognosis of 31 centromere and kinetochore protein genes using the R language and RNA profiles from The Cancer Genome Atlas (TCGA) RCC database. We found that the hazard ratio (HR) of CENPT ranked at the top five. The role of CENPT in cancers had not been previously elucidated. Previous studies had elucidated that the centromere and kinetochore gene CENP-T bound to the kinetochore throughout interphase and mitosis. Moreover, it formed complexes with other members (such as CENP-A/W), which functioned upstream of other components, and their depletion or deletion severely affected the recruitment of other centromere proteins (such as CENP-H/O/S) [39, 40]. Further research confirmed that CENPT was a key component in the assembly of the kinetochore protein complex, which was essential for normal mitotic progression. If CENPT was abnormal or its expression was reduced, mitosis was severely affected. Similarly, abnormalities in CENPT could directly lead to the disruption of the structure of the kinetochore protein complex (CENP-A NAC), resulting in mitotic errors and ultimately causing chromosomal aberrations [40]. This indicated that CENPT played an important role in maintaining normal cell function. However, little research had been conducted on other functions of CENPT, especially its relevance in tumors. Besides participating in mitosis, our study revealed a novel mechanism whereby CENPT enhanced the catalytic activity of GCLC by directly binding to GCLC in a competitive manner with GCLM. This interaction promoted the synthesis of GSH, inhibited ferroptosis, and ultimately drove the progression of RCC. Further research is required to determine whether the promoting effect of CENPT on RCC development was partly due to its involvement in mitosis.

We also found that the interaction between CENPT and GCLC increased the synthesis of GSH. This was consistent with previous studies that considerable level of GSH was elevated in GCLC overexpressing samples [41]. Previously, elevation of GCLC level was shown to promote GSH synthesis in a variety of diseases [42, 43]. ACTL6A increased the mRNA level of GCLC as a cotranscription factor of NRF2, thereby increasing GSH synthesis and promoting the progression of gastric cancer [44, 45]. GSH was known to be present in the most abundant non-protein in all mammalian tissues (thiol concentrations of 1–10 mM) and was not only resistant to oxidative stress, but also a key factor in redox signaling. On the other hand, it plays a crucial role in exogenous detoxification, regulation of cell proliferation, apoptosis, immune function, and fiber formation. In many tumors and even normal cell types, elevated glutathione levels are associated with cell proliferation and significantly correlate with cell cycle progression [46, 47]. To further validate the role of CENPT interaction with GCLC on RCC progression by affecting GSH expression, we tested this hypothesis through several experiments. Upregulation of CENPT was observed in cells treated with different doses of GSH. Tumor cell progression and drug resistance have been reported to be associated with elevated cellular GSH levels, GCL activity, GCLC gene transcription and mRNA levels [48, 49]. This was consistent with the findings of previous studies, which demonstrated that overexpression of GCLC increases GSH expression levels, playing a role in protecting cells from oxidation, resisting apoptosis, and even reducing radiation-induced cell death [50].

Furthermore, GSH synthesis was reduced after inhibition of GCLC expression, which typically has no effect on GCLM [43, 51]. Several studies have also shown that polymorphisms in the GCLC and GCLM genes exist in different populations [52, 53], which may account for differences in treatment sensitivity as well as efficacy among individuals with multiple diseases [50, 54, 55]. Moreover, studies have reported that GCLC mRNA levels and GSH expression levels were elevated in hepatocellular carcinoma cells, however, the expression of GCLM was not any altered in hepatocellular carcinoma cells [56]. Surprisingly, in our study, we found that the GCLM binding to GCLC was decreased by overexpression of CENPT in RCC cells. Meanwhile, our results showed that CENPT bound to GCLC ∆213-424aa, which domain was also required for GCLM binding. We then validated that the binding interaction of CENPT with GCLC might be in competition with GCLM by CO-IP assays. In addition, the expression levels of CENPT were increased by treatment with GSH, forming a CENPT-GCLC-GSH positive feedback loop to enhance the progression of RCC. It may be attributed to GSH increased the expression levels of CENPT via regulating some transcription factors. Importantly, our mechanistic studies found that ATF2 was a key transcription factor linking the GSH signal and CENPT’s transcriptional activation. Through chromatin immunoprecipitation and luciferase reporter assays, we demonstrated that ATF2 directly bound to the E3 motif (−1200 to −1184 bp) in the CENPT promoter- a regulatory element uniquely responsive to GSH-mediated induction. This discovery aligned with ATF2’s established role as a redox-sensitive transcription factor. ATF2 could integrate environmental cues (such as GSH levels) to regulate gene expression programs [57], and had participated in cell proliferation, survival, and metastasis in various tumors by regulating target genes [58–60]. Notably, the E3 binding site resided in the proximal promoter region, indicating ATF2 served as a proximal regulator of CENPT transcription. Furthermore, the absence of GSH-induced CENPT upregulation in ATF2-knockdown cells highlighted the transcription factor’s indispensability in mediating the GSH-CENPT axis. As a core transcription factor responding to oxidative stress, ATF2 integrated redox signals from GSH, forming a GSH-ATF2-CENPT regulatory axis to precisely control CENPT expression, thereby influencing RCC progression. This mechanism not only clarified the transcriptional regulation basis for CENPT’s upregulated expression in tumors but also revealed how tumor cells leverage the interaction network of redox signals and transcription factors to coordinate gene expression and promote proliferation. This is different from GSH negative inhibition feedback to inhibit GCLC activity under normal physiological conditions [17]. All in all, CENPT-GCLC-GSH positive feedback loop gave us an improved understanding of the role played by GSH in cancer microenvironment.

**Fig. 7:**
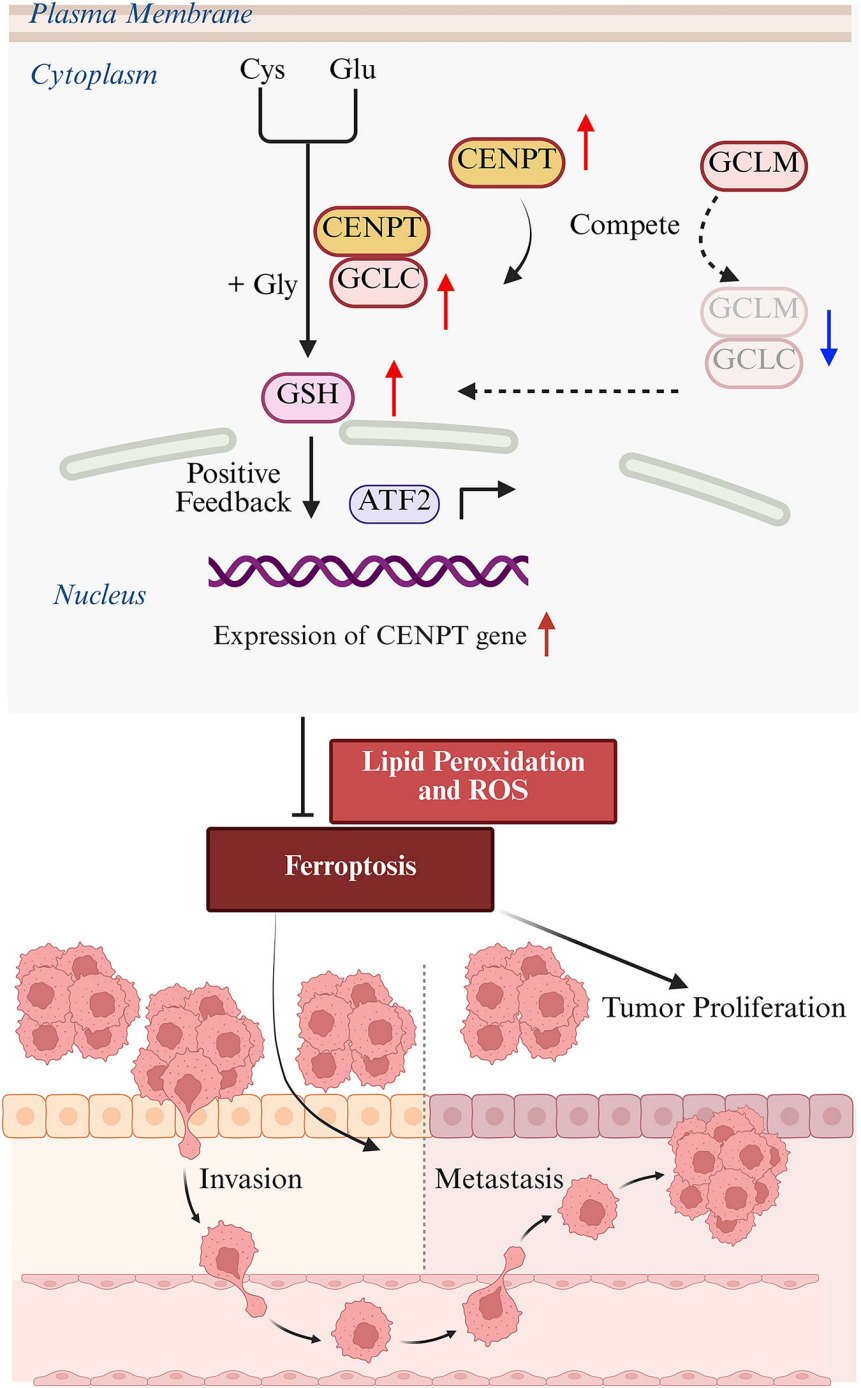
Schematic diagram of CENPT mediating GSH synthesis and inhibiting ferroptosis. CENPT interacts with GCLC by competing with GCLM to promote GSH synthesis and inhibit ferroptosis in RCC cells. In turn, GSH increases the expression level of CENPT at transcript level via activation the transcription factor ATF2, forming a CENPT-GCLC-GSH feedback loop that enhances the pro-carcinogenic effect of this axis in RCC cells.

GSH is an antioxidant that shields cancer cells from oxidative stress through its synthesis cycle, thereby reducing intracellular ROS levels and promoting tumor growth [61]. We validated the impact of CENPT-GCLC on GSH synthesis, resulting in a decrease in lipid peroxidation and ROS levels, mediating RCC ferroptosis resistance and proliferation. Ferroptosis is a form of programmed cell death characterized by ROS and lipid peroxidation, triggered when the antioxidant status is compromised [62]. Increasing evidence suggests that GCLC inhibits ferroptosis by increasing GSH levels [45, 63]. Our data showed that treatment with the ferroptosis inhibitor Fer-1 reduced shCENPT-induced cell death, suggesting that ferroptosis contributes to shCENPT-induced RCC cell death. Furthermore, after treating the cells with the ferroptosis activator erastin, the inhibitory effect of CENPT on ferroptosis was reversed, and an impact on RCC progression and metastasis was observed. This confirmed the relevance of the CENPT-GCLC-GSH signaling axis in the ferroptosis pathway. It’s noteworthy that ferroptosis involves various metabolic processes and also influences responses to cancer chemotherapy, radiotherapy, and immunotherapy [64, 65]. Therefore, targeting the CENPT-GCLC-GSH signaling axis to induce ferroptosis is a promising potential strategy for RCC treatment (Fig. 7).

Together, this study uncovered a GSH-driven regulatory axis in RCC progression, where CENPT acts as both a functional effector and a transcriptional target. First, we demonstrated CENPT’s role in regulating GCLC and GSH production, and identified its mechanism of promoting RCC progression by reducing ROS levels and lipid peroxidation, thereby inhibiting ferroptosis. Second, we showed that accumulating GSH activates ATF2 to transcriptionally amplify CENPT expression. This self-reinforcing loop promotes RCC proliferation and ferroptosis resistance through ROS and lipid peroxidation reduction. Importantly, this framework bridges antioxidant metabolism with transcriptional regulation, offering a novel perspective on how tumors exploit redox homeostasis for malignant progression. Targeting CENPT represents a promising therapeutic strategy for RCC.

## Supplementary information

Supplementary Material

uncropped westernblots qPCR

## Data Availability

Data of this study are available upon request from the authors.
