# Supplementary material for: CENPT prevents renal cell carcinoma against ferroptosis by enhancing the synthesis of glutathione
Source: Cell Death Dis. 2025 Jul 12;16(1):517. doi: 10.1038/s41419-025-07848-x (PMC12255702; doi:10.1038/s41419-025-07848-x)
Supplement: Supplementary file 1 — Supplementary Material [file 41419_2025_7848_MOESM1_ESM.docx]

***Supplementary material***

**CENPT Prevents Renal Cell Carcinoma against Ferroptosis by Enhancing the Synthesis of Glutathione**

Han Yang^1,^ *, Zongliang Zhang^1,^ *, Ninghan Feng^2, 3,^ *, Kai Zhao^1^, Yulian Zhang^4^, Xinbao Yin^1^, Guanqun Zhu^1^, Zhenlin Wang^1^, Xuechuan Yan^1^, Xueyu Li^1^, Zhaofeng Li^1^, Qinglei Wang^1^, Yixin Qi^1^, Peng Zhao^5,^ **^†^**, Tianzhen He^6,^ **^†^**, Ke Wang^1,^ **^†^**

1. Department of Urology, The Affiliated Hospital of Qingdao University, Qingdao, Shangdong, China

2. Department of Urology, Jiangnan University Medical Center, Wuxi, China

3. Department of Urology, Wuxi No.2 Hospital, Nanjing Medical University, Wuxi, China

4. Department of Gynecology, The Affiliated Hospital of Qingdao University, Qingdao, Shangdong, China

5. Faculty of Sport Science and Coaching, Universiti Pendidikan Sultan Idris, Tanjong Malim, Perak Darul Ridzuan, 35900, Malaysia

6. Nantong University, Institute of special environmental medicine, Nantong, 226019, China

* These authors contributed equally to this work.

**^†^**Correspondence to:

Ke Wang. E-mail: wangke@qdu.edu.cn

Tianzhen He. E-mail: sailing198562@ntu.edu.cn

Peng Zhao. E-mail: peng.zhao@dukekunshan.edu.cn

**Table S1.** Primer sequences for qPCR.

| **Primer** | | **Sequence** |
| --- | --- | --- |
| CENPT | Forward | GGTGAGAGGATTGTCAACCAG |
|  | Reverse | AGTCTTGGCTTCTTCATCATATTCT |
| GCLC | Forward | ACTTCATTTCCCAGTACCTTAACA |
|  | Reverse | GAAATCACTCCCCAGCGACA |
| ATF2 | Forward | AATTGAGGAGCCTTCTGTTGTAG |
|  | Reverse | CATCACTGGTAGTAGACTCTGGG |
| GAPDH | Forward | GGAAGCTTGTCATCAATGGAAATC |
|  | Reverse | TGATGACCCTTTTGGCTCCC |

**Table S2.** Potential ATF2 interacting motifs in the 5′-upstream flanking region of the CENPT gene.

| **Location (relative to TSS)** | **Sequence (5′ to 3′)** | **Orientation** |
| --- | --- | --- |
| L1 (from 1884 to 1896 +) | atgaagtcat | F |
| L2 (from 1155 to 1164 +) | atgatctcag | F |
| L3 (from 587 to 596 -) | atgaggacat | F |

**Table S3.** The potential cancer prognosis of 31 centromere and kinetochore protein genes.

**
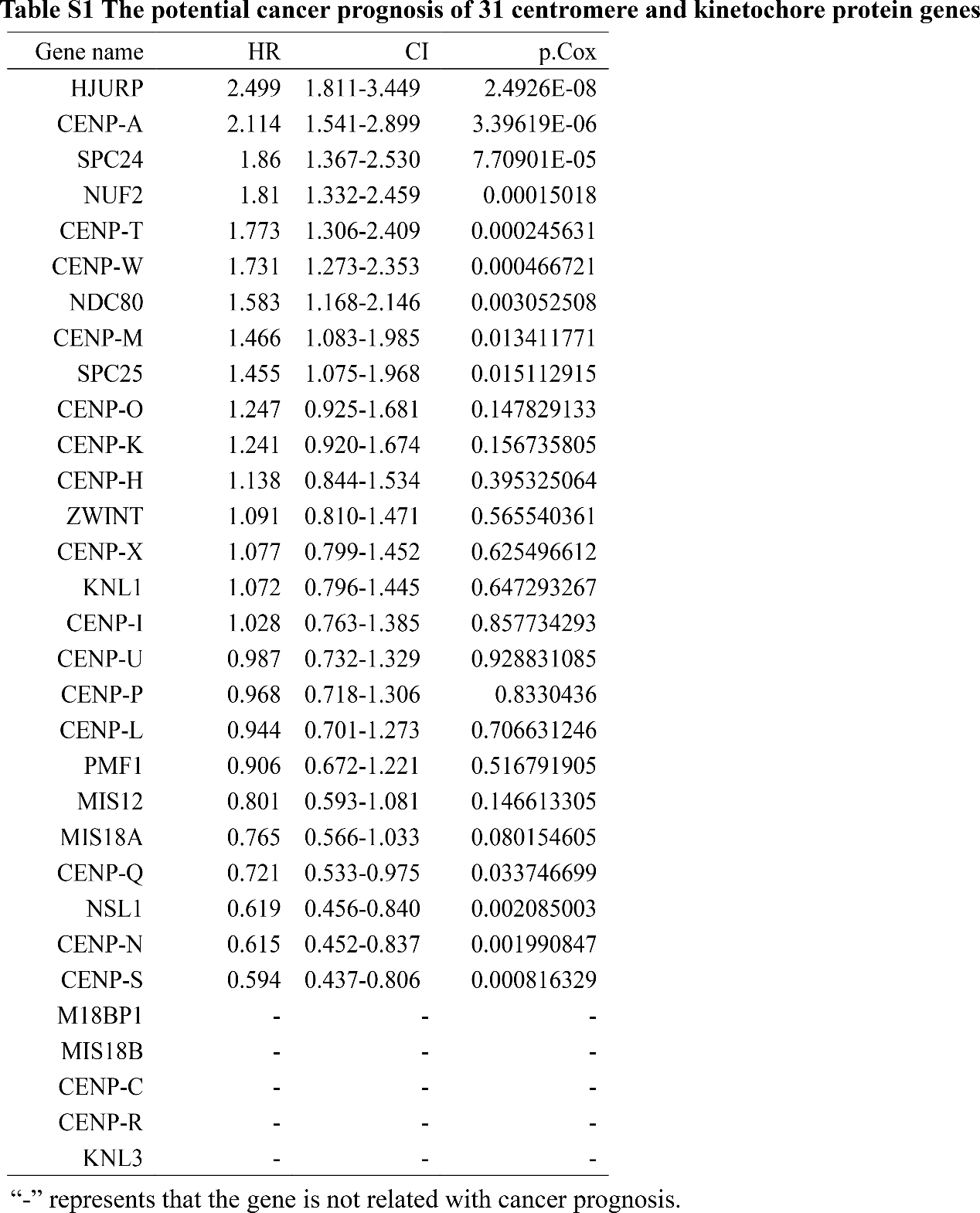
**

**Table S4.** CENPT different lentiviral shRNA oligonucleotide sequences.

| **Gene** | **Gene ID** | **TargetSeg** | **Outcome** |
| --- | --- | --- | --- |
| CENPT | NM_025082.4 | TGGAGAAGTGCCTAGATAAAT | shCENPT-1 |
| CENPT | NM_025082.4 | GCCACTATGTGAAACTCTTTA | shCENPT-2 |
| NC2 | NA | CCTAAGGTTAAGTCGCCCTCG | shNC |

**fig. S1**


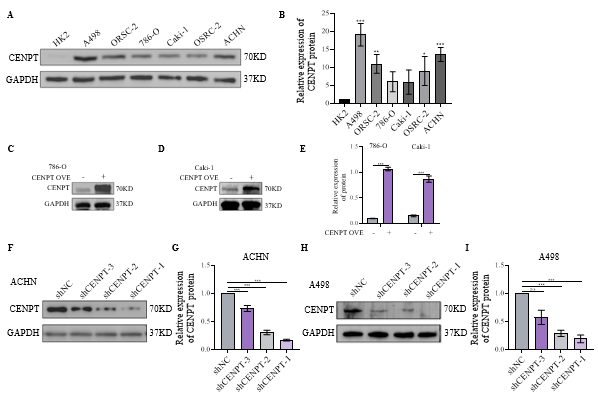


**The expression of CENPT in different RCC cells and the knockdown efficiency of CENPT shRNA in transfected RCC cells.** (A-B) The relative expression of CENPT in different RCC cells. (C-E) The overexpression efficiency of CENPT in 786-O and Caki-1 cells was validated by WB. (F-I) The knockdown efficiency of CENPT shRNA was validated by WB. The data are presented as the means ± SEM (n=3). Compared with the indicated groups, *p < 0.05, **p < 0.01, ***p < 0.001. ns, no significant difference.

**fig S2**

**
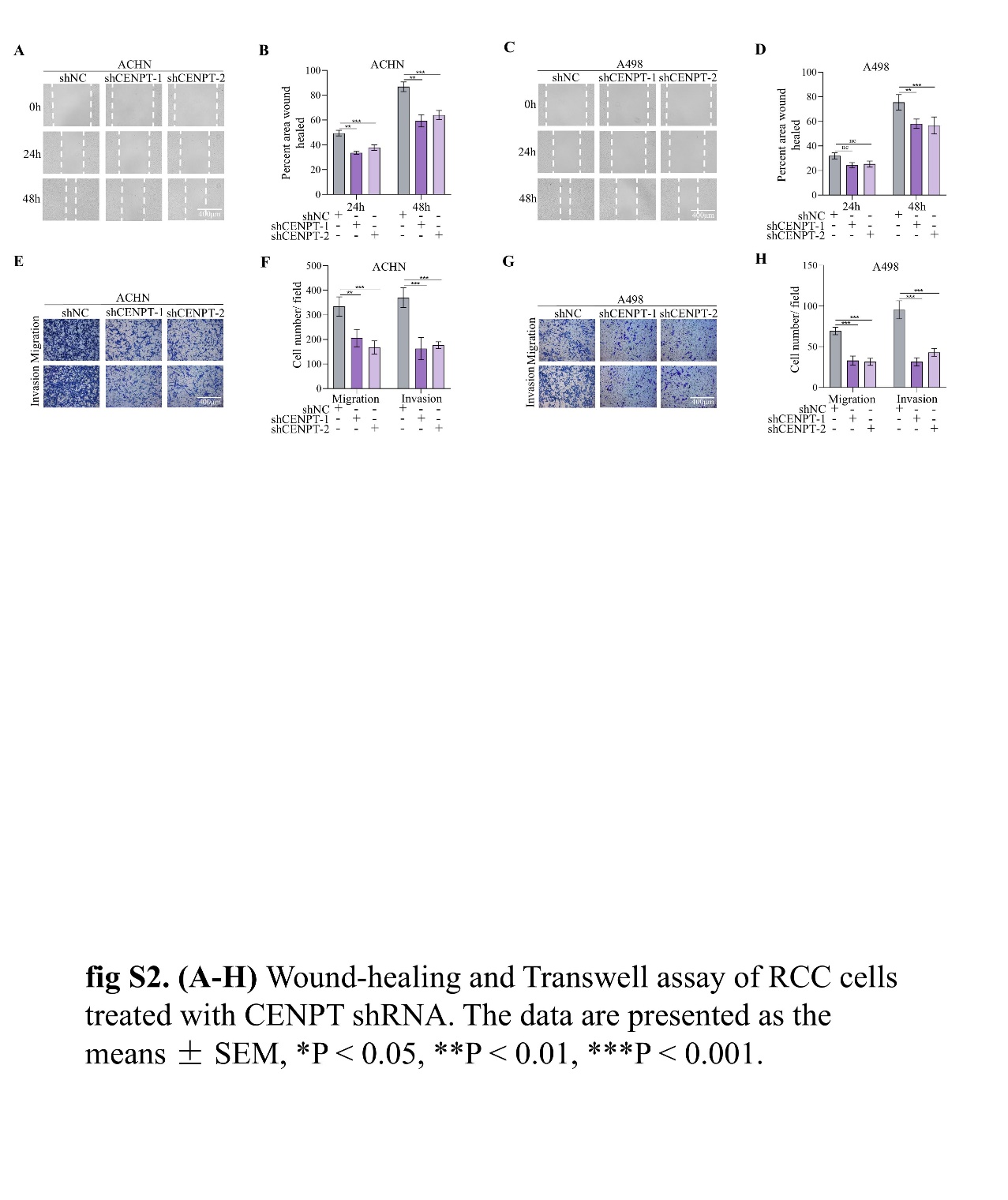
**

**CENPT shRNAs inhibited the migration and invasion of RCC cells.** (A-H) Wound-healing and Transwell assay of RCC cells treated with CENPT shRNA. The data are presented as the means ± SEM (n=3). Compared with the indicated groups, *p < 0.05, **p < 0.01, ***p < 0.001.


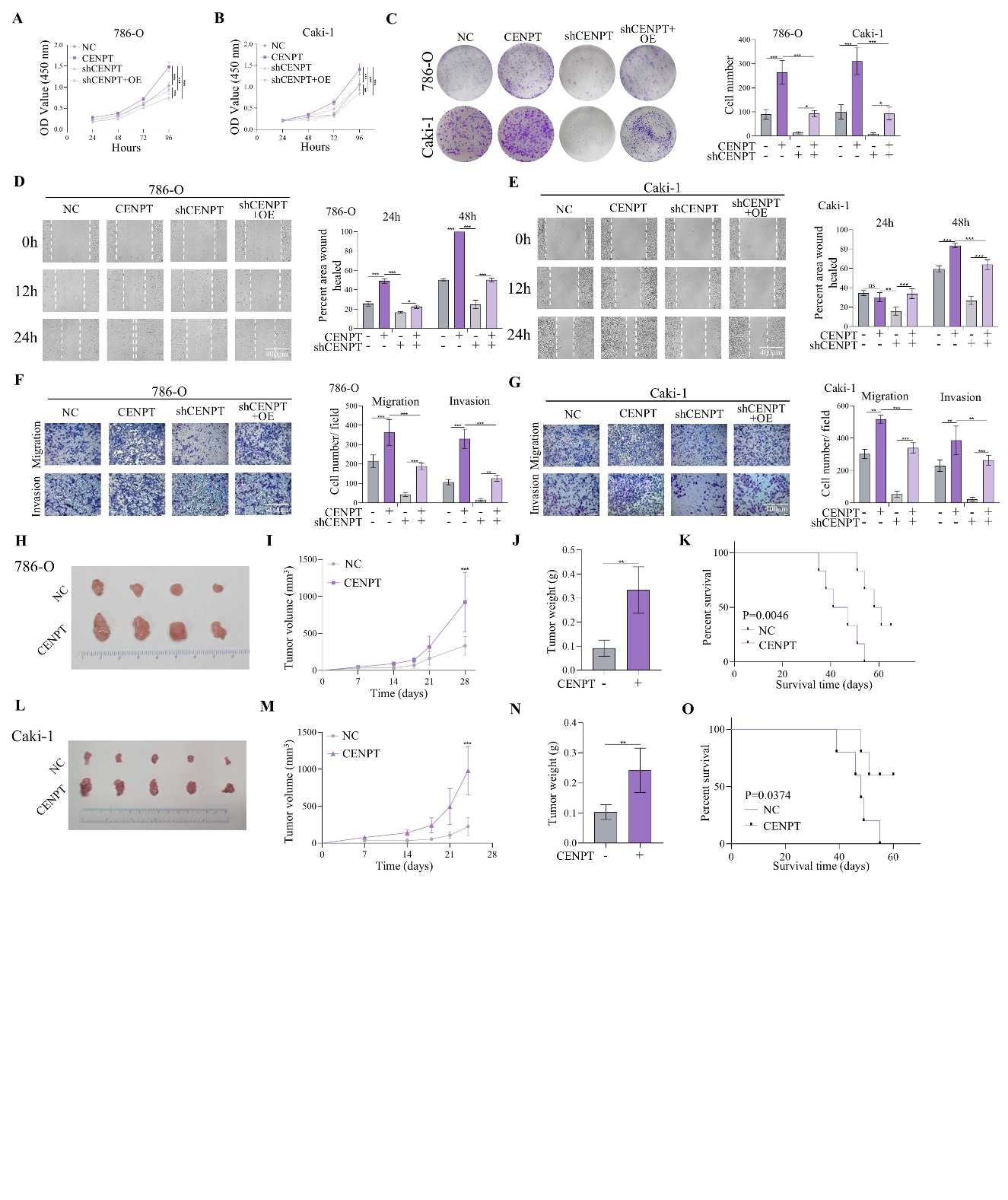
**fig S3**

**shRNA-mediated silencing of CENPT expression inhibited proliferation, migration and invasion of RCC cells.** (A-B) CCK-8 assay analyzing cell proliferation in different cells. (C) Colony formation assay assessing cell proliferative ability in different cells. (D-E) Cell migratory ability was assessed by wound healing assay in different cells. (F-G) Transwell assay assessing cell invasion ability in different cells. (H/L) Gross image of subcutaneous tumors derived from a xenograft model using different RCC cells. (I/M) Tumor growth curve of different groups. (J/N) The tumor weights of 786-O and Caki-1 cells stably transfected with NC or CENPT OVE. (K/O) Mice survival curves (n = 5 or n=6). (P-Q) ROS levels in RCC cells after CENPT knockdown. Data are given as mean ± SEM.*P < 0.05, **P < 0.01, ***P < 0.001 (Student's t-test).

**fig S4**

**
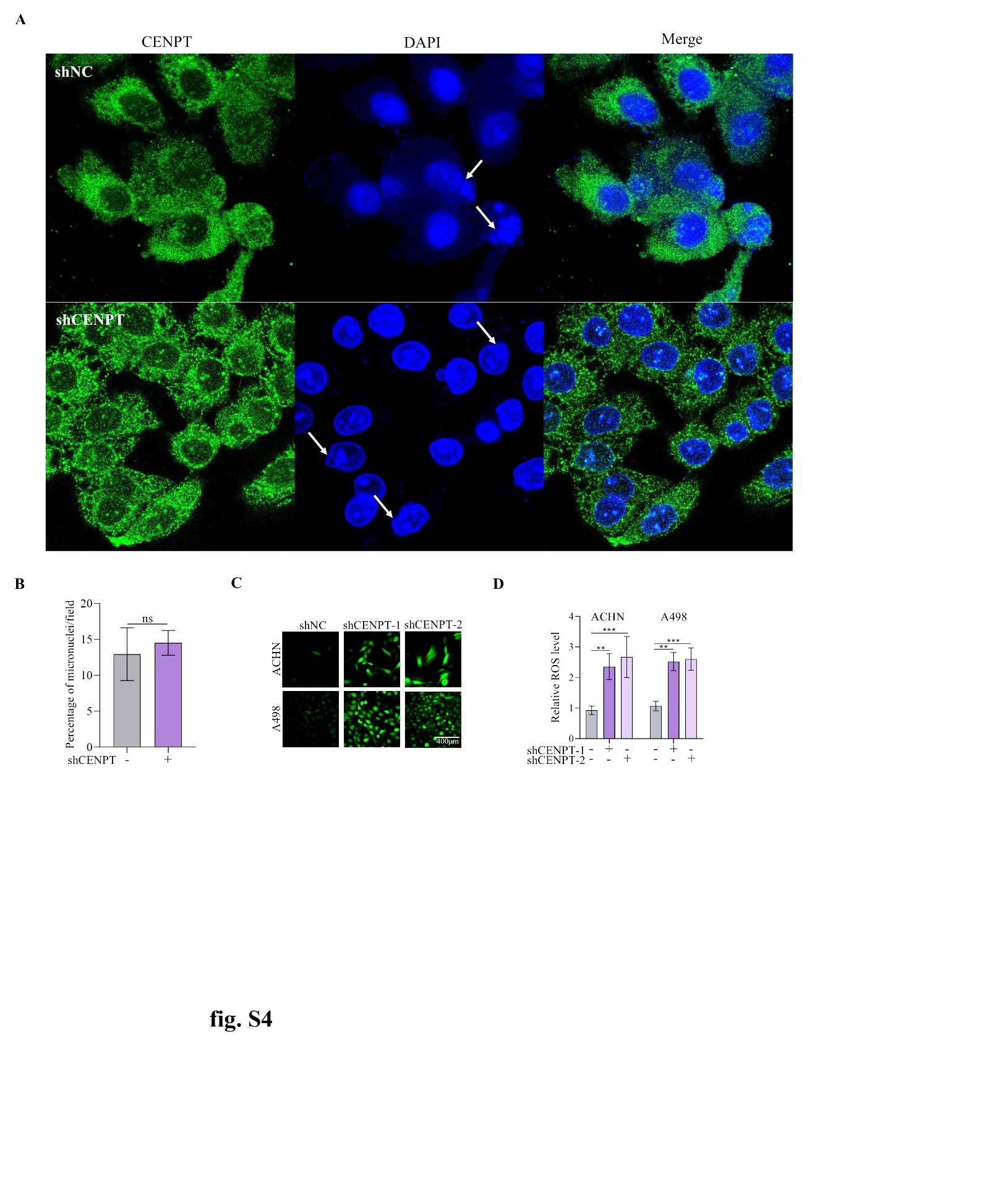
**

(A-B) Immunostaining was performed using an anti-CENPT antibody (green) and DAPI. Representative images of micronuclei/multinucleated cells (marked by white arrowheads) in shNC- and shCENPT-transfected RCC cells. (C-D) ROS levels in RCC cells after CENPT knockdown. The data are presented as the means ± SEM, *P < 0.05, **P < 0.01, ***P < 0.001 (Student's t-test).

**fig S5**


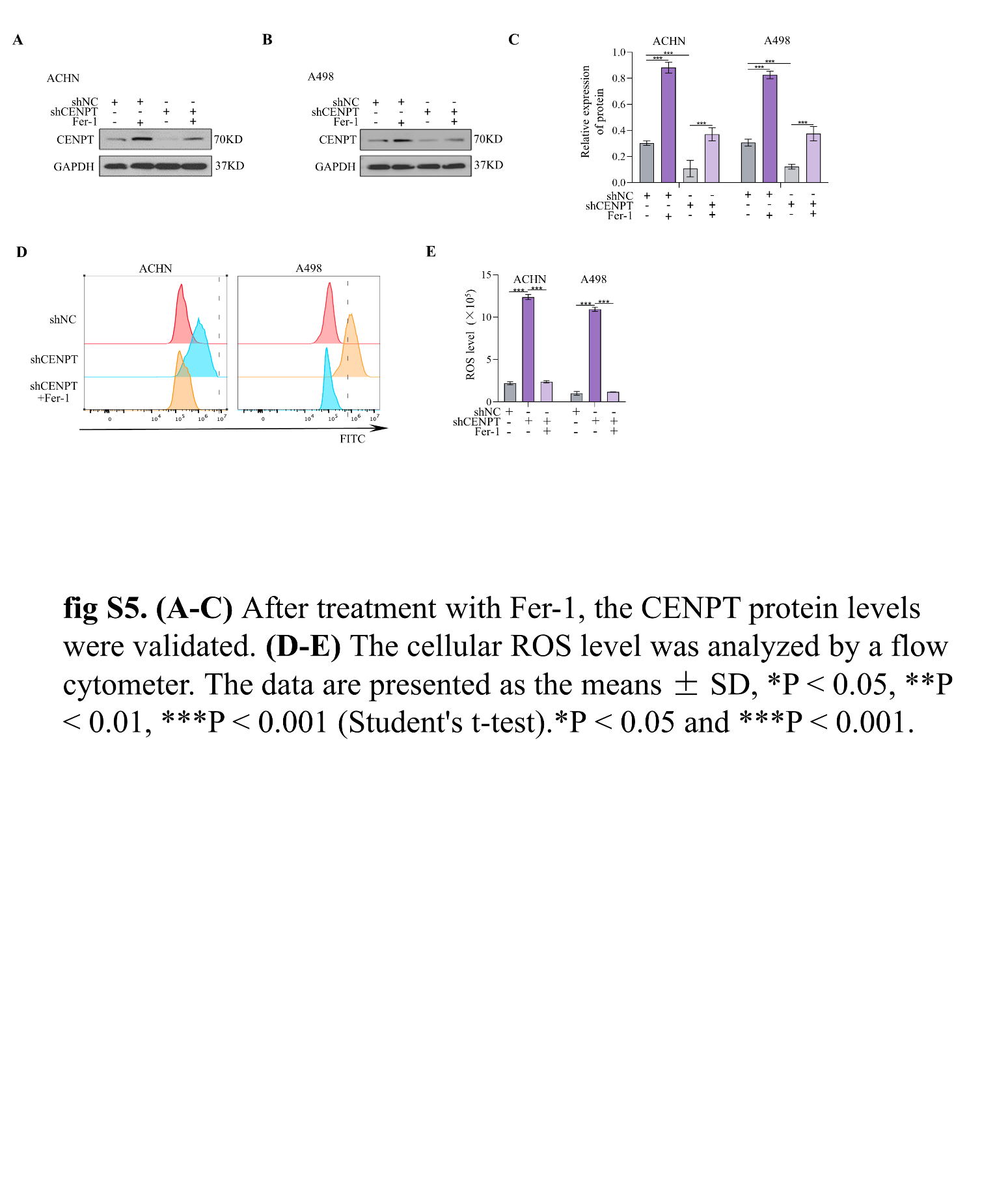


**The expression level of CENPT was decreased by treatment with Fer-1.** (A-C) After treatment with Fer-1, the CENPT protein levels were validated. The data are presented as the means ± SEM, *P < 0.05, **P < 0.01, ***P < 0.001 (Student's t-test).

**fig S6**


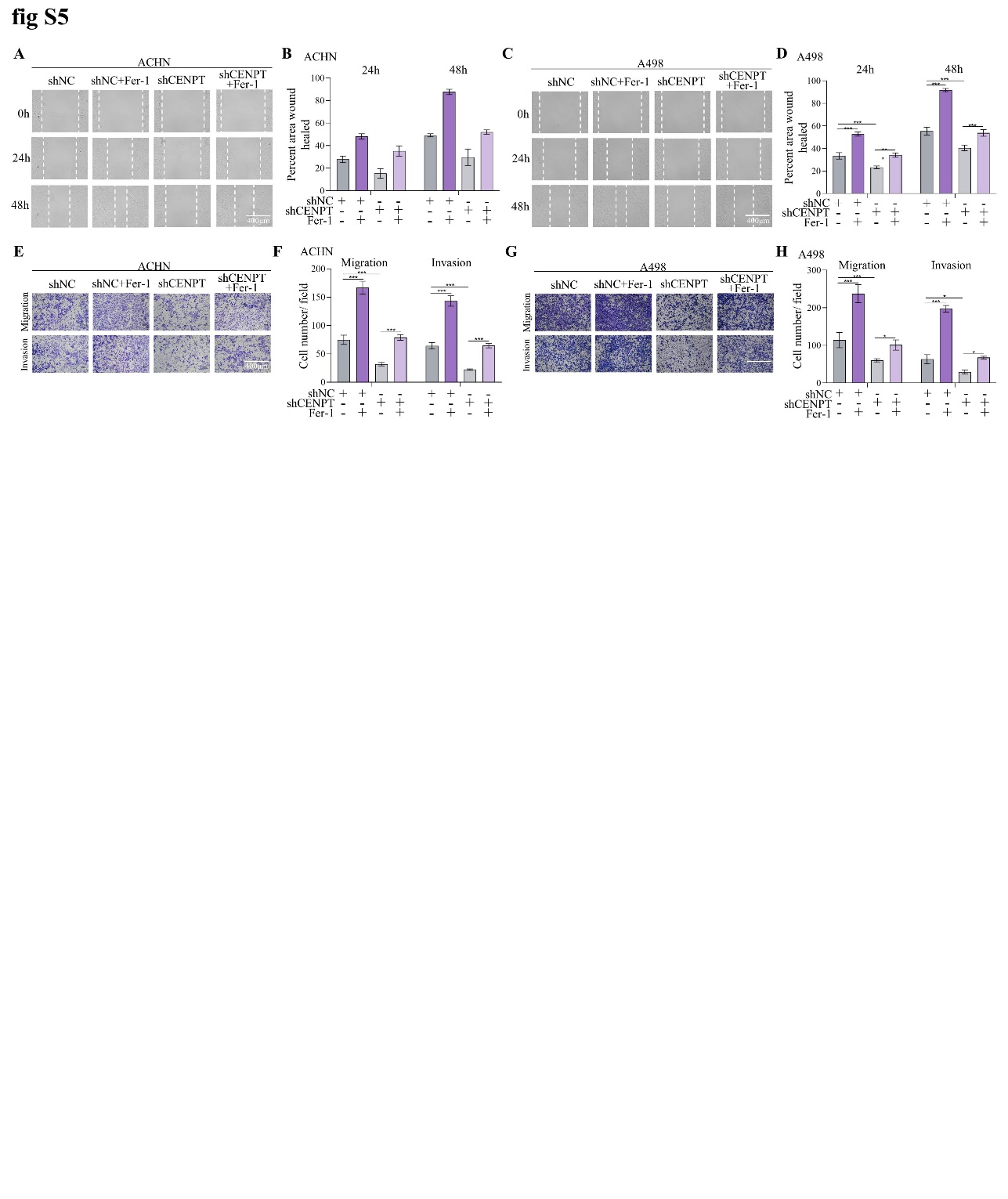


**The inhibitory effect of shCENPT on the growth of RCC cells was reversed by treatment with ferroptosis inhibitor Fer-1.** (A-H) After treatment with Fer-1, different cell lines underwent wound healing assays and migration and invasion capability analyses. The data are presented as the means ± SEM (n=3). Compared with the indicated groups, *p < 0.05, **p < 0.01, ***p < 0.001.

**fig S7**


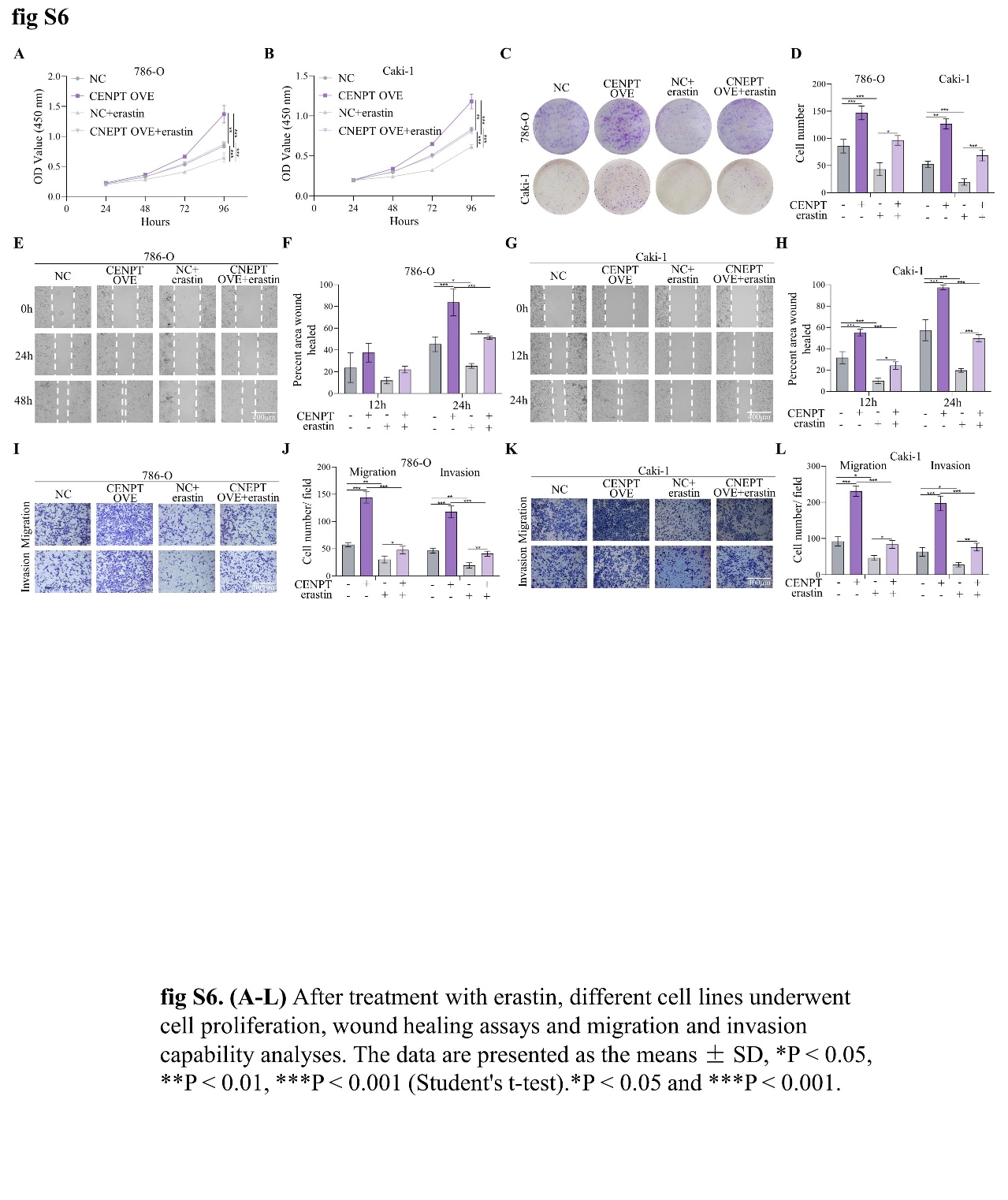


**After treatment with erastin, different cell lines underwent cell proliferation, wound healing assays and migration and invasion capability analyses** (A-L). The data are presented as the means ± SEM, *P< 0.05**P<0.01, ***P<0.001 (Student's t-test).

**
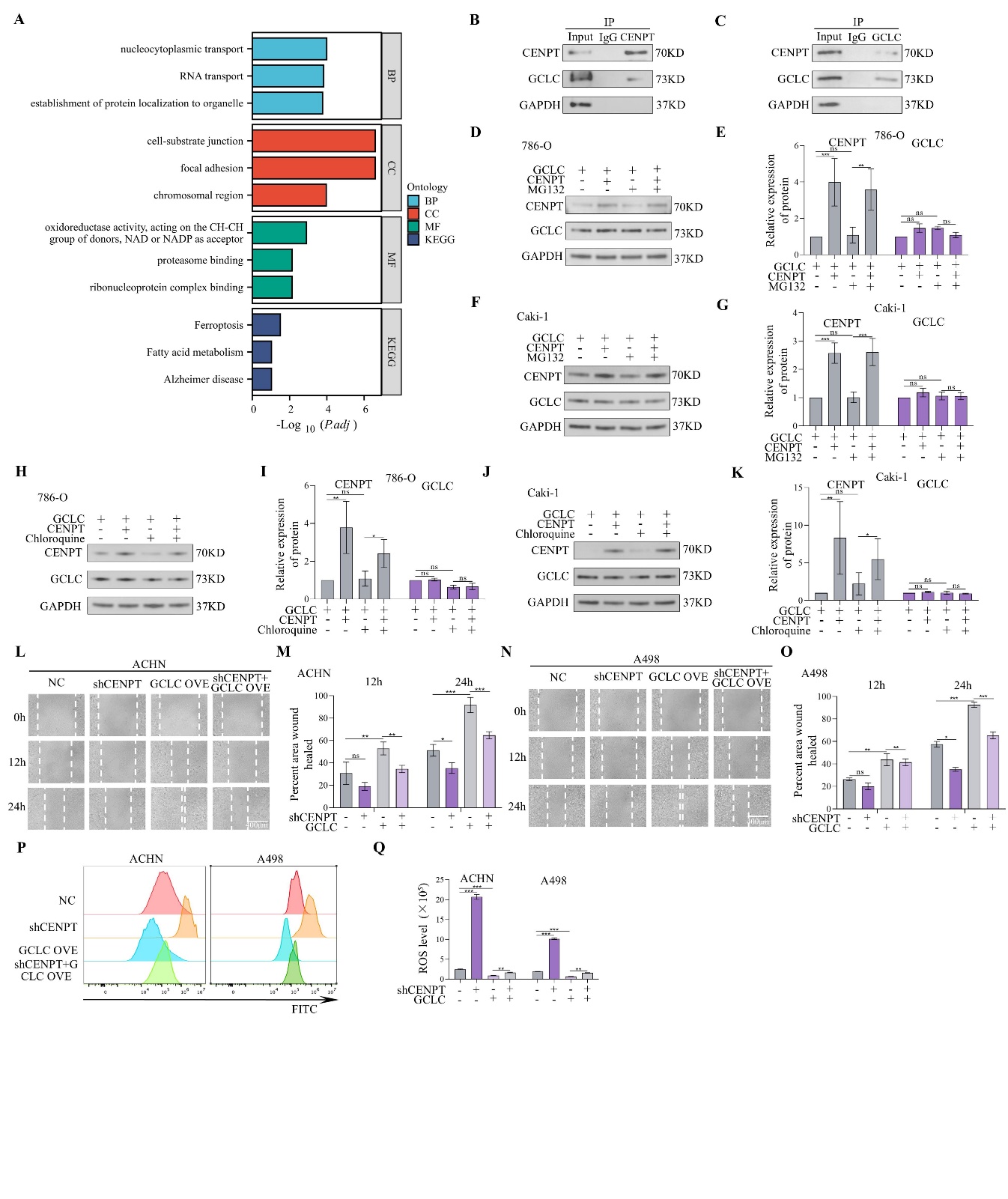
fig S8**

**CENPT was the binding partner of GCLC.** (A) The associated proteins were further enrichment analyzed. (B-I) Protein levels of CENPT and GCLC in RCCs after treatment with MG132 or chloroquine. (J-M) Representative results of wound healing assays in ACHN and A498 cells showed that overexpression of GCLC significantly restored the migratory capability of RCC cancer cells against CENPT shRNAs. (N-O) The cellular ROS level was analyzed by flow cytometer. Data are given as mean ± SEM. *P < 0.05, **P < 0.01, ***P < 0.001 (Student's t-test).

**fig S9**


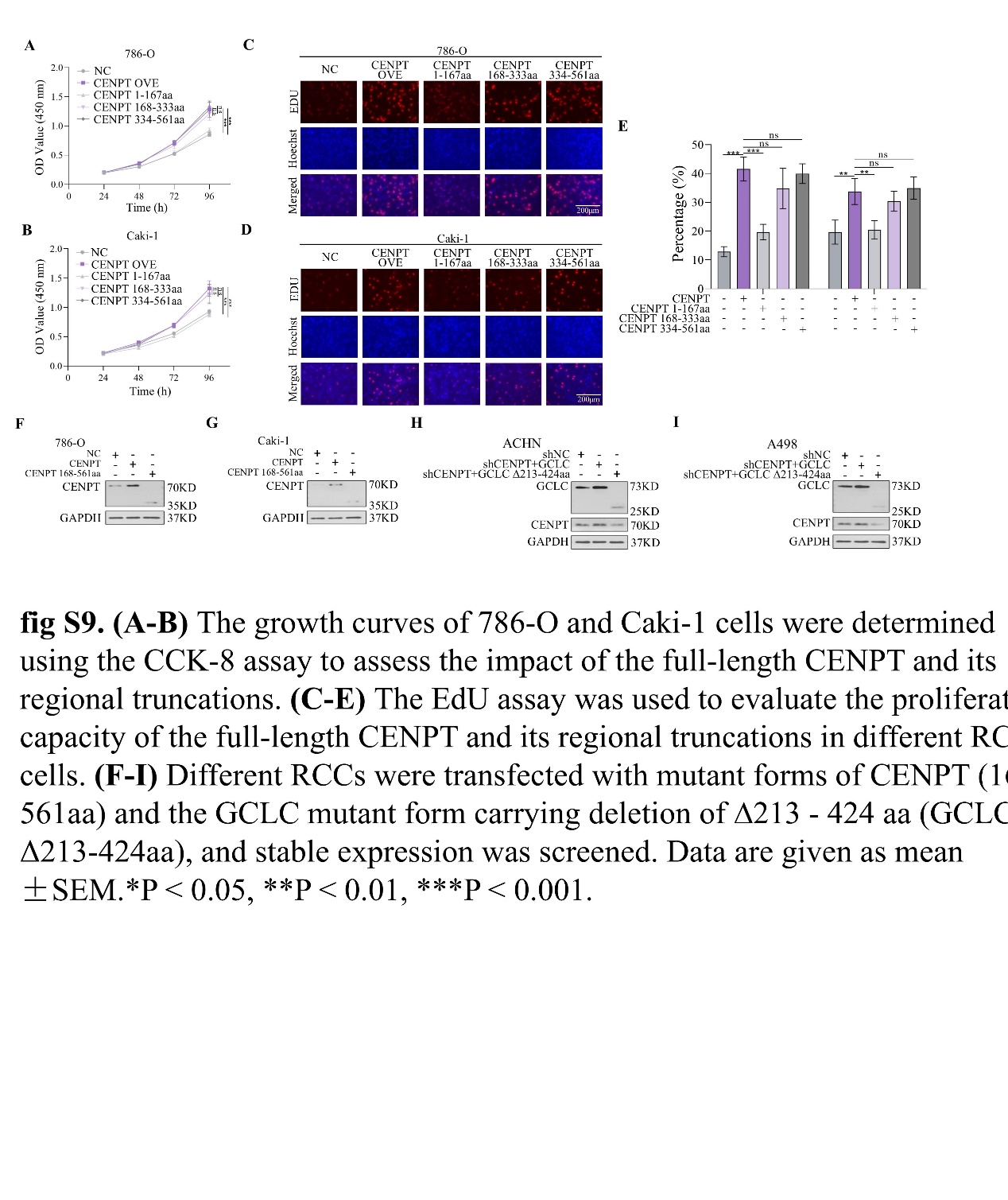


**The promotion of RCC development is mediated by the interaction between the ∆N terminal region of CENPT and GCLC.** (A-B) The growth curves of 786-O and Caki-1 cells were determined using the CCK-8 assay to assess the impact of the full-length CENPT and its regional truncations. (C-E) The EdU assay was used to evaluate the proliferative capacity of the full-length CENPT and its regional truncations in different RCC cells. (F-I) Different RCCs were transfected with mutant forms of CENPT (168-561aa) and the GCLC mutant form carrying deletion of 213-424 aa (GCLC △213-424aa), and stable expression was screened. Data are given as mean ±SEM.*P < 0.05, **P < 0.01, ***P < 0.001.

**fig S10**


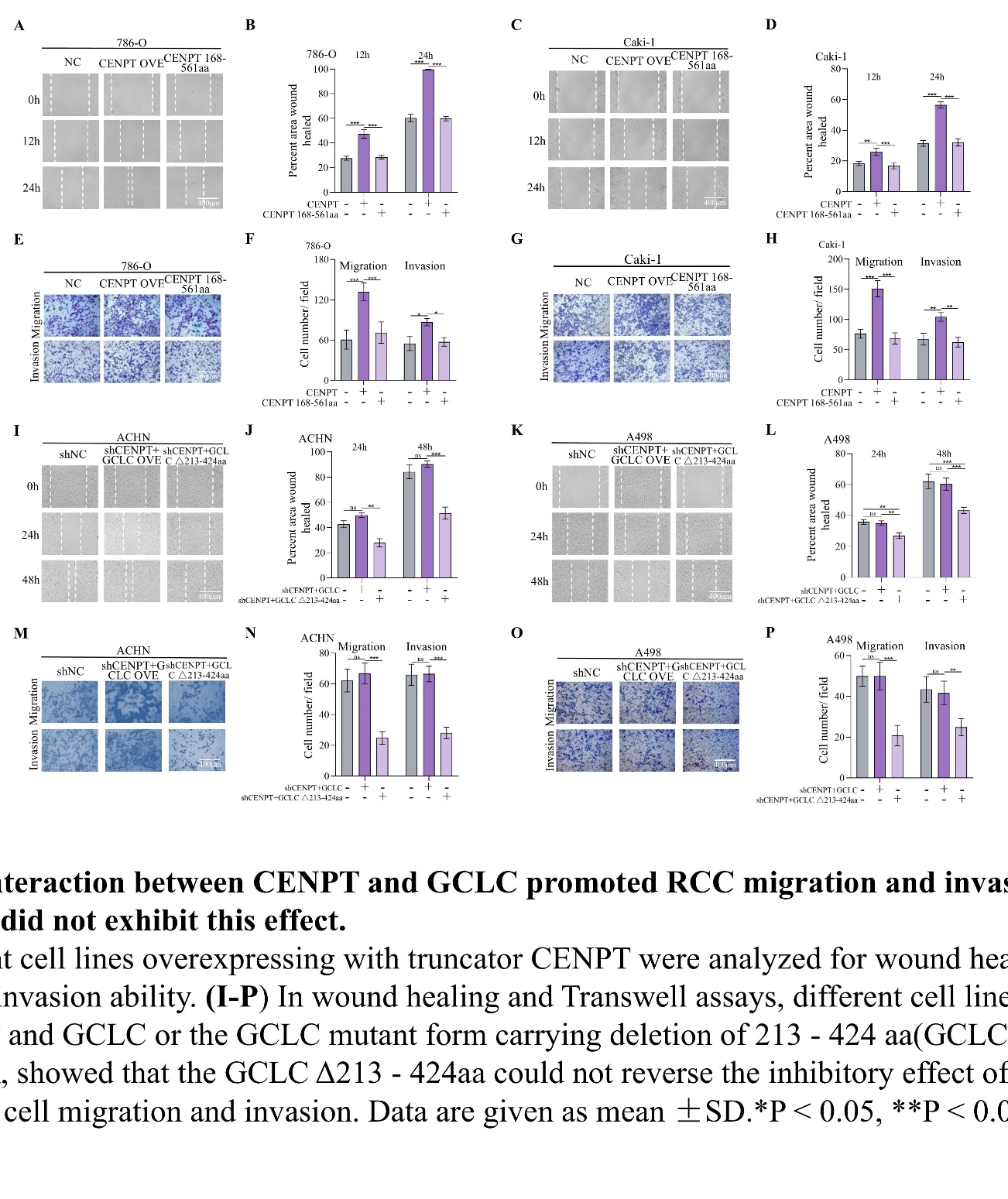


**The truncations of CENPT and GCLC didn’t promoted RCC migration and invasion *in vitro*.** (A-H) Different cell lines overexpressing with CENPT truncator were analyzed for wound healing assay and migration and invasion ability. (I-P) In wound healing and transwell assays, different cell lines co - transfected with shCENPT and GCLC or the GCLC mutant form carrying deletion of 213-424 aa (GCLC △213-424aa) overexpression, showed that the GCLC △213-424aa could not reverse the inhibitory effect of CENPT knockdown on cell migration and invasion. Data are given as mean ± SEM (n=3). Compared with the indicated groups, *p < 0.05, **p < 0.01, ***p < 0.001.

**fig S11**

**
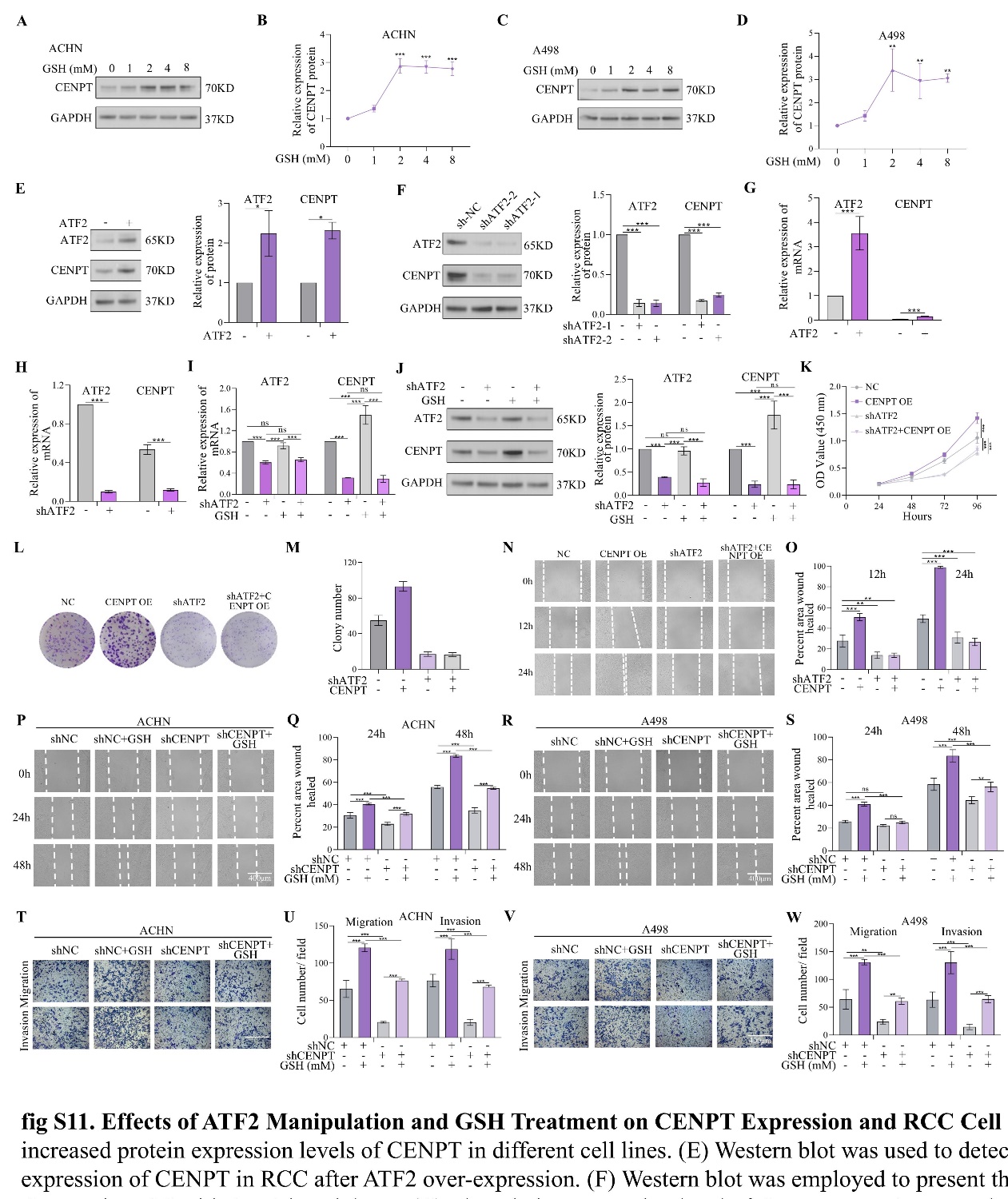
**

**Effects of ATF2 Manipulation and GSH Treatment on CENPT Expression and RCC Cell Behavior.** (A-D) GSH increased protein expression levels of CENPT in different cell lines. (E) Western blot was used to detect and show the protein expression of CENPT in RCC after ATF2 over-expression. (F) Western blot was employed to present the protein expression of CENPT in RCC with ATF2 knockdown. (G) The relative expression level of CENPT mRNA was analyzed after stable ATF2 over-expression. (H) In the samples with shATF2 and control samples, the mRNA expression of CENPT was significantly decreased. (I-J) The relative expression level of CENPT mRNA and protein was analyzed in RCC with shATF2 and GSH treatment. (K-O) Proliferation and migration abilities of RCC in different treatment groups (NC, CENPT overexpression, shATF2, shATF2 + CENPT overexpression). (P-S) Wound healing experiments evaluated the migratory ability of ACHN and A498 cells with CENPT knockdown after GSH treatment. (T-W) Transwell assays were used to measure the migration and invasion capabilities of RCC cells with CENPT knockdown in the presence of GSH. Data are given as mean ±SEM (n=3). Compared with the indicated groups, *p < 0.05, **p < 0.01, ***p < 0.001.
