# Supplementary material for: CENPT prevents renal cell carcinoma against ferroptosis by enhancing the synthesis of glutathione
Source: Cell Death Dis. 2025 Jul 12;16(1):517. doi: 10.1038/s41419-025-07848-x (PMC12255702; doi:10.1038/s41419-025-07848-x)
Supplement: Supplementary file 2 — uncropped westernblots qPCR [file 41419_2025_7848_MOESM2_ESM.docx]

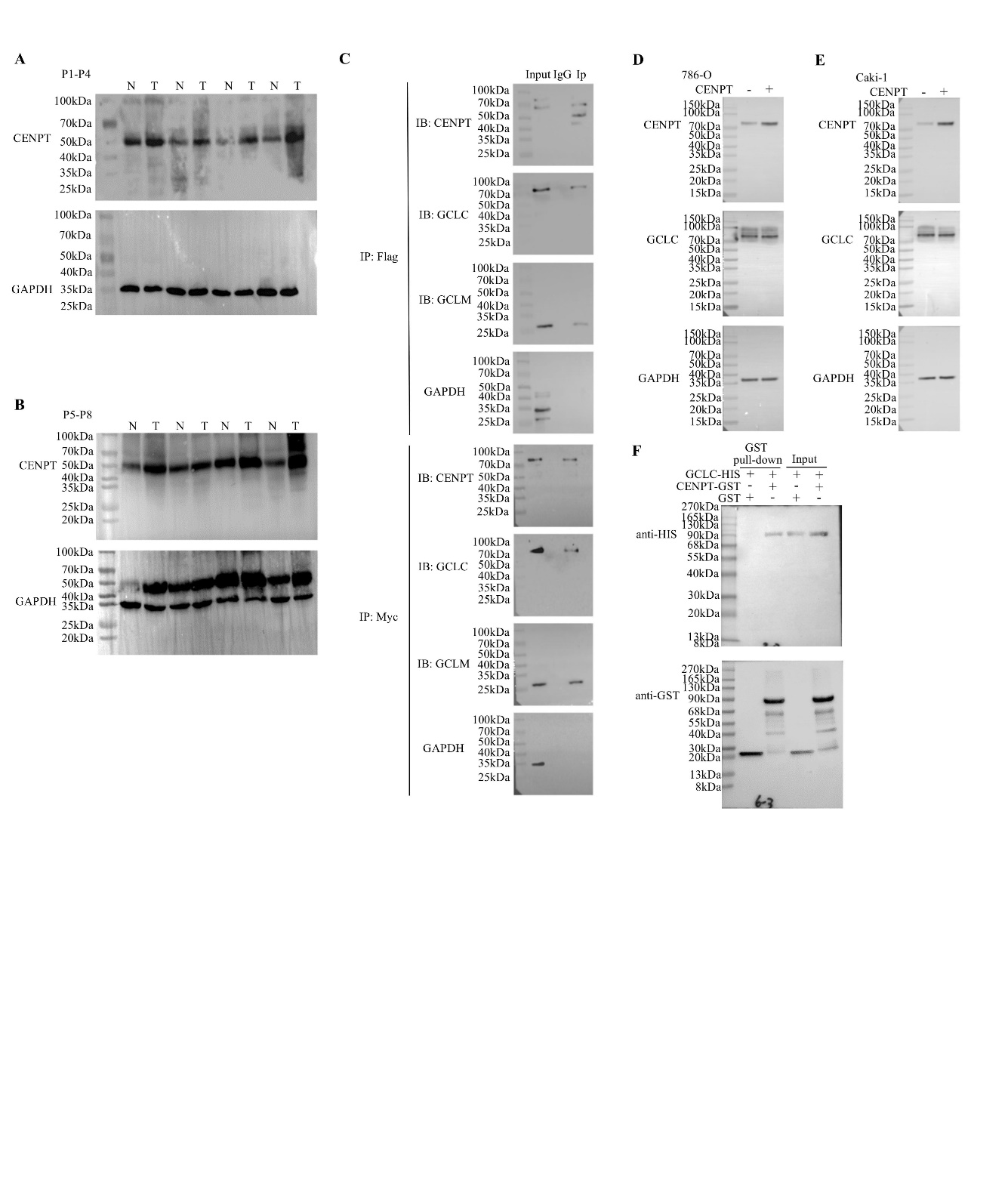


**Raw Data of WB.** (A-B) Raw data western blot Fig 1F. (C) Raw data western blot Fig 4A. (D-E) Raw data western blot Fig 4B. (F) Raw data western blot Fig 4D.


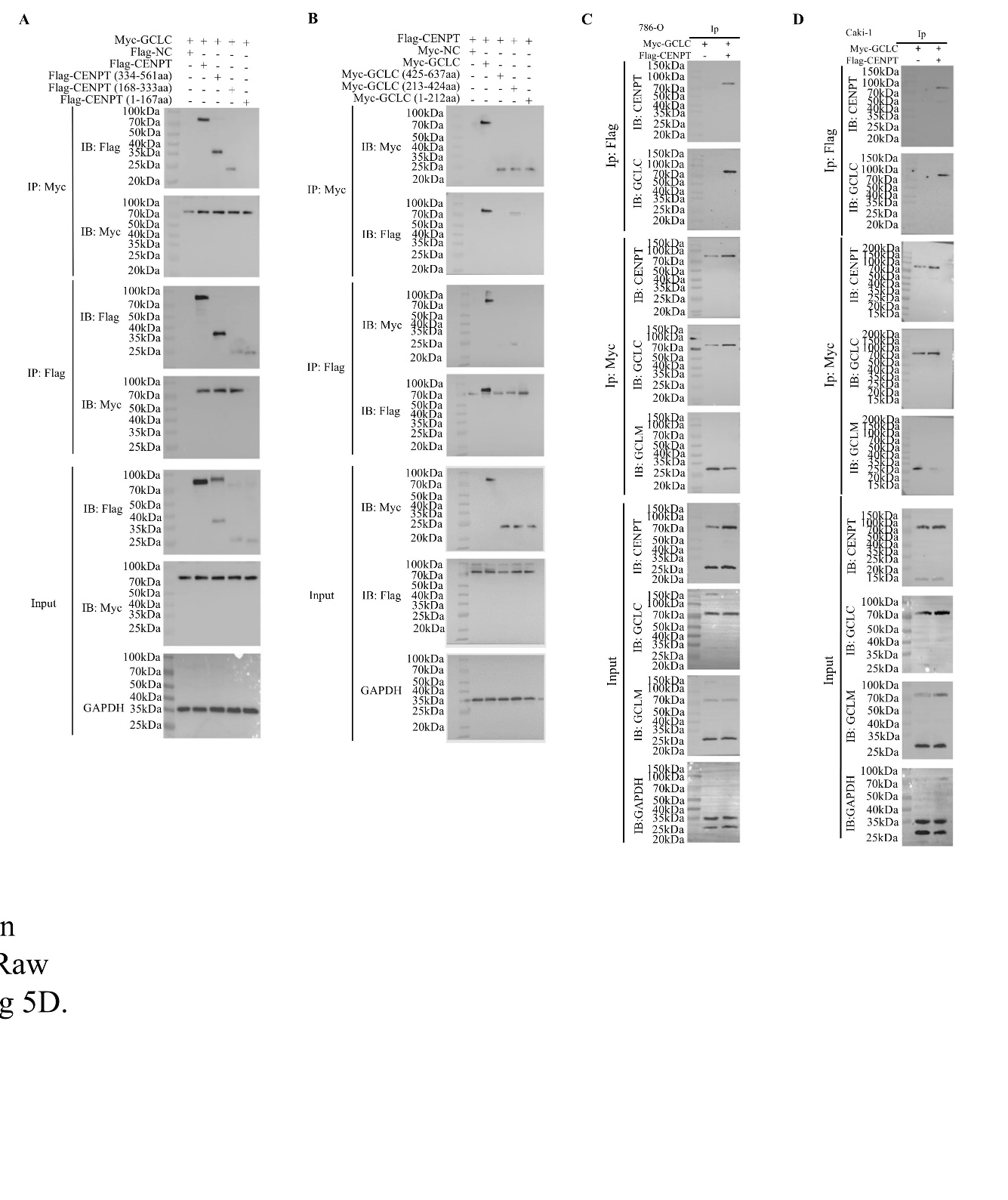


**Raw Data of WB.** (A-B) Raw data western blot Fig 5A and Fig 5B. (C-D) Raw data western blot Fig 5C and Fig 5D.


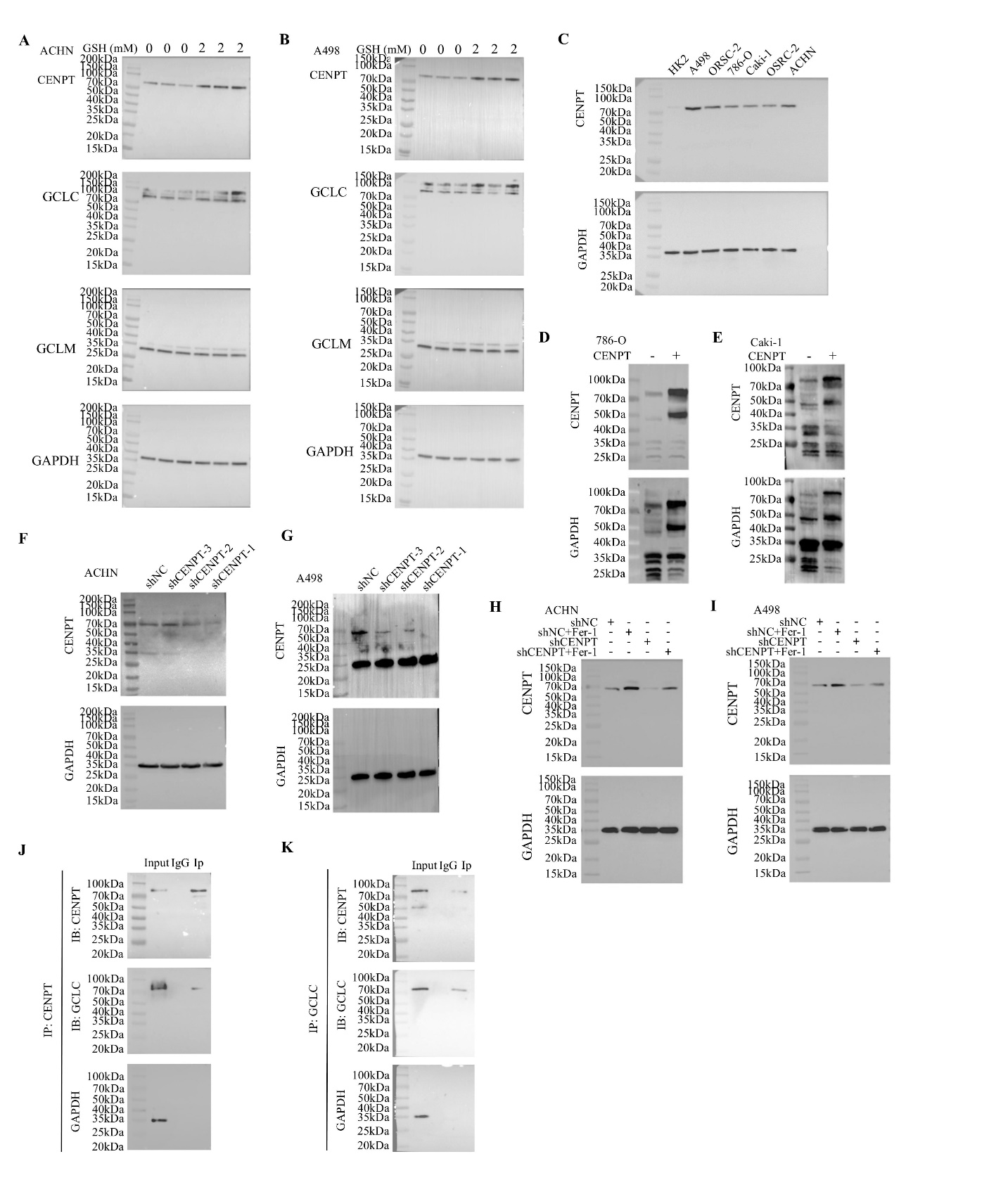


**Western blot raw data.** (A-B) Raw data western blot Fig 6H and Fig 6I. (C) Raw data western blot fig S1A. (D-E) Raw data western blot fig S1C and fig S1D. (F-G) Raw data western blot fig S1F and fig S1H. (H-I) Raw data western blot fig S5A and fig S5B. (J-K) Raw data western blot fig S8B and fig S8C.


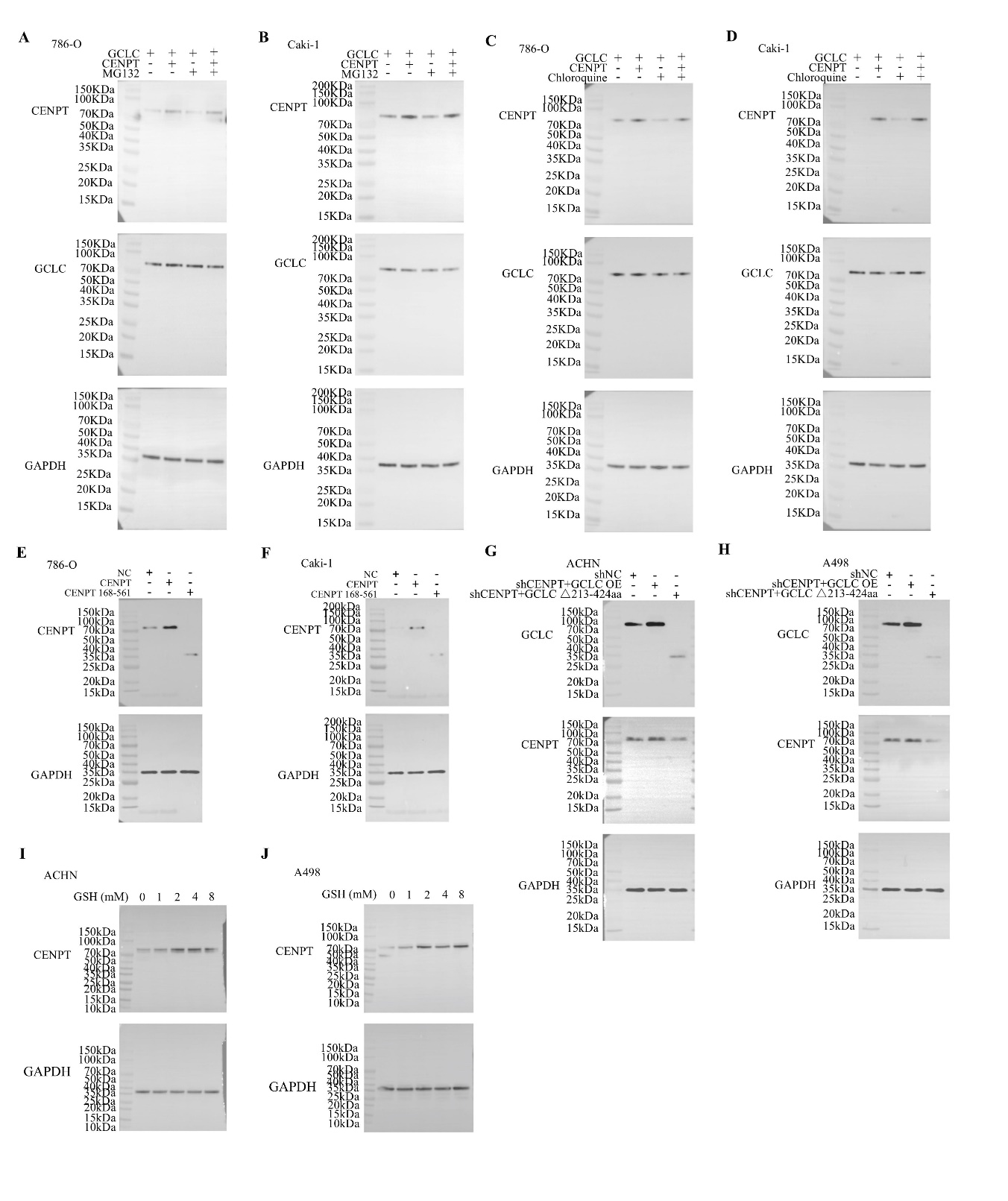


**Western blot raw data.** (A-B) Raw data western blot fig S8D and fig S8F. (C-D) Raw data western blot fig S8H and fig S8J. (E-F) Raw data western blot fig. S9F and fig. S9G. (G-H) Raw data western blot fig. S9H and fig S9I. (I-J) Raw data western blot fig. S11A and fig S11C.


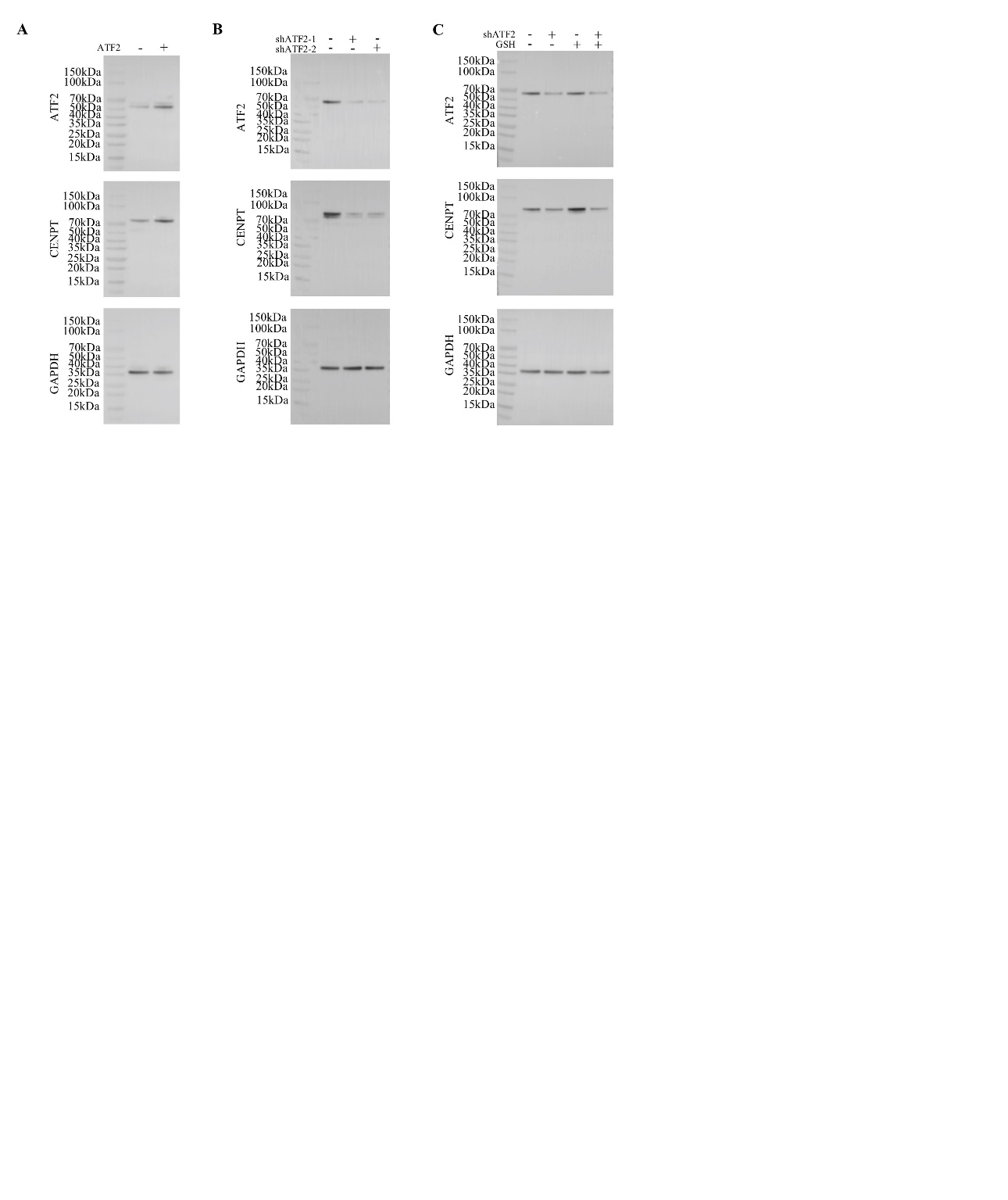


**Western blot raw data.** (A-B) Raw data western blot fig S11E and fig S11F. (C) Raw data western blot fig S11J.

| Well | Target | Sample | Cq |
| --- | --- | --- | --- |
| A01 | CENPT | 1A | 20.06 |
| A02 | CENPT | 1A | 20.16 |
| A03 | CENPT | 1A | 20.12 |
| A04 | CENPT | 2A | 30.25 |
| A05 | CENPT | 2A | 31.26 |
| A06 | CENPT | 2A | 30.98 |
| A07 | CENPT | 3A | 27.82 |
| A08 | CENPT | 3A | 27.75 |
| A09 | CENPT | 3A | 27.91 |
| A10 | CENPT | 4A | 29.87 |
| A11 | CENPT | 4A | 30.15 |
| B01 | CENPT | 5A | 31.09 |
| B02 | CENPT | 5A | 31.75 |
| B03 | CENPT | 5A | 32.07 |
| B04 | CENPT | 6A | 23.29 |
| B05 | CENPT | 6A | 23.46 |
| B06 | CENPT | 6A | 23.42 |
| B07 | CENPT | 7A | 31.93 |
| B08 | CENPT | 7A | 31.18 |
| B09 | CENPT | 7A | 32.49 |
| B10 | CENPT | 8A | 30.44 |
| B11 | CENPT | 8A | 30.61 |
| B12 | CENPT | 8A | 30.61 |
| C01 | CENPT | 9A | 27.73 |
| C02 | CENPT | 9A | 27.60 |
| C03 | CENPT | 9A | 27.46 |
| C04 | CENPT | 10A | 30.90 |
| C05 | CENPT | 10A | 30.72 |
| C06 | CENPT | 10A | 31.11 |
| C07 | CENPT | 1T | 20.80 |
| C08 | CENPT | 1T | 20.83 |
| C09 | CENPT | 1T | 20.97 |
| C10 | CENPT | 2T | 26.98 |
| C11 | CENPT | 2T | 27.07 |
| C12 | CENPT | 2T | 27.09 |
| D01 | CENPT | 3T | 28.28 |
| D02 | CENPT | 3T | 27.98 |
| D03 | CENPT | 3T | 28.03 |
| D04 | CENPT | 4T | 21.60 |
| D05 | CENPT | 4T | 21.74 |
| D06 | CENPT | 4T | 21.55 |
| D07 | CENPT | 5T | 22.90 |
| D08 | CENPT | 5T | 23.00 |
| D09 | CENPT | 5T | 23.12 |
| D10 | CENPT | 6T | 21.69 |
| D11 | CENPT | 6T | 21.81 |
| D12 | CENPT | 6T | 21.80 |
| Well | Target | Sample | Cq |
| D1 | CENPT | NC | 30.23 |
| D2 | CENPT | NC | 30.21 |
| D3 | CENPT | NC | 29.97 |
| D4 | CENPT | OE | 27.3 |
| D5 | CENPT | OE | 27.18 |
| D6 | CENPT | OE | 27.07 |
| E1 | GAPDH | NC | 18.33 |
| E2 | GAPDH | NC | 18.18 |
| E3 | GAPDH | NC | 18.91 |
| E4 | GAPDH | OE | 17.62 |
| E5 | GAPDH | OE | 18.6 |
| E6 | GAPDH | OE | 18.33 |
| F1 | GCLC | NC | 28.11 |
| F2 | GCLC | NC | 28.21 |
| F3 | GCLC | NC | 28.34 |
| F4 | GCLC | OE | 28.08 |
| F5 | GCLC | OE | 28.19 |
| F6 | GCLC | OE | 28.43 |
| G1 | GAPDH | NC | 17.34 |
| G2 | GAPDH | NC | 17.81 |
| G3 | GAPDH | NC | 17.24 |
| G4 | GAPDH | OE | 17.56 |
| G5 | GAPDH | OE | 17.83 |
| G6 | GAPDH | OE | 18.04 |
| Well | Target | Sample | Cq |
| A7 | CENPT | NC | 30.99 |
| A8 | CENPT | NC | 31.51 |
| A9 | CENPT | NC | 30.98 |
| A10 | CENPT | GSH | 28.21 |
| A11 | CENPT | GSH | 28.05 |
| A12 | CENPT | GSH | 28.09 |
| B7 | GAPDH | NC | 15.84 |
| B8 | GAPDH | NC | 15.84 |
| B9 | GAPDH | NC | 15.86 |
| B10 | GAPDH | GSH | 15.73 |
| B11 | GAPDH | GSH | 15.68 |
| B12 | GAPDH | GSH | 15.68 |
| Well | Target | Sample | Cq |
| C10 | ATF2 | NC | 27.33 |
| C11 | ATF2 | NC | 27.45 |
| C12 | ATF2 | NC | 27.31 |
| D10 | GAPDH | NC | 15.65 |
| D11 | GAPDH | NC | 15.7 |
| D12 | GAPDH | NC | 15.62 |
| E10 | ATF2 | OE | 25.87 |
| E11 | ATF2 | OE | 25.25 |
| E12 | ATF2 | OE | 25.93 |
| F10 | GAPDH | OE | 15.77 |
| F11 | GAPDH | OE | 15.77 |
| F12 | GAPDH | OE | 15.73 |
| G7 | CENPT | NC | 31.75 |
| G8 | CENPT | NC | 32.11 |
| G9 | CENPT | NC | 31.88 |
| G10 | CENPT | OE | 29.25 |
| G11 | CENPT | OE | 29.49 |
| G12 | CENPT | OE | 29.52 |
| H7 | GAPDH | NC | 15.7 |
| H8 | GAPDH | NC | 15.68 |
| H9 | GAPDH | NC | 15.68 |
| H10 | GAPDH | OE | 14.92 |
| H11 | GAPDH | OE | 15.04 |
| H12 | GAPDH | OE | 15.01 |
| Well | Target | Sample | Cq |
| A1 | ATF2 | NC | 26.64 |
| A2 | ATF2 | NC | 26.44 |
| A3 | ATF2 | NC | 26.45 |
| A4 | CENPT | NC | 28.05 |
| A5 | CENPT | NC | 28.05 |
| A6 | CENPT | NC | 28.11 |
| A7 | CENPT | sh | 30.85 |
| A8 | CENPT | sh | 30.76 |
| A9 | CENPT | sh | 30.96 |
| B1 | GAPDH | NC | 16.33 |
| B2 | GAPDH | NC | 16.43 |
| B3 | GAPDH | NC | 16.35 |
| B4 | GAPDH | NC | 17.03 |
| B5 | GAPDH | NC | 17.03 |
| B6 | GAPDH | NC | 17.01 |
| B7 | GAPDH | sh | 17.63 |
| B8 | GAPDH | sh | 17.67 |
| B9 | GAPDH | sh | 17.61 |
| C1 | ATF2 | sh | 28.52 |
| C2 | ATF2 | sh | 27.96 |
| C3 | ATF2 | sh | 27.99 |
| D1 | GAPDH | sh | 14.65 |
| D2 | GAPDH | sh | 14.73 |
| D3 | GAPDH | sh | 14.68 |
| Well | Target | Sample | Cq |
| E1 | ATF2 | shNC | 26.5 |
| E2 | ATF2 | shNC | 26.34 |
| E3 | ATF2 | shNC | 26.49 |
| E4 | ATF2 | shNC+G | 27.26 |
| E5 | ATF2 | shNC+G | 27.26 |
| E6 | ATF2 | shNC+G | 27.26 |
| F1 | GAPDH | shNC | 14.8 |
| F2 | GAPDH | shNC | 14.84 |
| F3 | GAPDH | shNC | 14.81 |
| F4 | GAPDH | shNC+G | 15.53 |
| F5 | GAPDH | shNC+G | 15.57 |
| F6 | GAPDH | shNC+G | 15.49 |
| G1 | ATF2 | sh | 28.45 |
| G2 | ATF2 | sh | 28.46 |
| G3 | ATF2 | sh | 28.34 |
| G4 | ATF2 | sh+G | 28.65 |
| G5 | ATF2 | sh+G | 28.59 |
| G6 | ATF2 | sh+G | 28.57 |
| H1 | GAPDH | sh | 16.04 |
| H2 | GAPDH | sh | 16.05 |
| H3 | GAPDH | sh | 16.07 |
| H4 | GAPDH | sh+G | 16.17 |
| H5 | GAPDH | sh+G | 16.73 |
| H6 | GAPDH | sh+G | 16.19 |
| E7 | CENPT | shNC | 29.63 |
| E8 | CENPT | shNC | 29.33 |
| E9 | CENPT | shNC | 29.53 |
| E10 | CENPT | shNC+G | 26.8 |
| E11 | CENPT | shNC+G | 26.84 |
| E12 | CENPT | shNC+G | 26.82 |
| F7 | GAPDH | shNC | 16.13 |
| F8 | GAPDH | shNC | 16.16 |
| F9 | GAPDH | shNC | 16.09 |
| F10 | GAPDH | shNC+G | 14.07 |
| F11 | GAPDH | shNC+G | 14.02 |
| F12 | GAPDH | shNC+G | 13.99 |
| G7 | CENPT | sh | 31.26 |
| G8 | CENPT | sh | 30.87 |
| G9 | CENPT | sh | 31.08 |
| G10 | CENPT | sh+G | 31.18 |
| G11 | CENPT | sh+G | 31.49 |
| G12 | CENPT | sh+G | 31.13 |
| H7 | GAPDH | sh | 16.02 |
| H8 | GAPDH | sh | 16.02 |
| H9 | GAPDH | sh | 16.06 |
| H10 | GAPDH | sh+G | 16.12 |
| H11 | GAPDH | sh+G | 16.09 |
| H12 | GAPDH | sh+G | 16.1 |
